# A Semantic-Enhanced Multi-Source Fusion Localization Method for GNSS-Degraded Environments

**DOI:** 10.3390/s26123761

**Published:** 2026-06-12

**Authors:** Haobo Zhao, Xinhua Tang

**Affiliations:** School of Instrument Science and Engineering, Southeast University, Nanjing 210096, China; 220233710@seu.edu.cn

**Keywords:** semantic information, multi-source integrated positioning, factor graph optimization, simultaneous localization and mapping, unmanned ground vehicle

## Abstract

In complex urban environments, Global Navigation Satellite System (GNSS) signals are easily affected by building blockage and multipath effects, which may degrade positioning quality or even cause GNSS denial. As a result, conventional integrated navigation systems suffer from accumulated errors due to insufficient global constraints. To address this problem, a multi-source integrated positioning method incorporating semantic information is proposed. Fixed traffic lights are selected as semantic landmarks, and an object detection network is used to extract the center pixel coordinates and detection confidence of the landmarks. Then, by combining depth information, camera pose, and the prior global coordinates of fixed semantic landmarks, a semantic target inversion model is established to transform two-dimensional image information into three-dimensional position estimates in the world coordinate system. Semantic factors are further constructed and incorporated into backend factor graph optimization. To determine the weighting of semantic factors, the influences of pixel localization error, depth estimation error, camera pose error, and prior coordinate error of fixed semantic landmarks on semantic observations are analyzed, and a noise covariance model for semantic factors is established. Finally, an unmanned ground vehicle experimental platform is built to validate and analyze the proposed factor graph algorithm. The experimental results show that, under GNSS-degraded conditions, the algorithm with semantic factors can provide supplementary global constraints for the system and effectively suppress accumulated positioning errors. In Experiment 1, compared with the algorithm without semantic factors, the maximum absolute trajectory error is reduced by 46.26%. To further verify the applicability of the proposed method in more complex scenarios, Experiment 2 is conducted on a longer route with multiple semantic landmarks and a more severe GNSS-degraded interval. The results show that the proposed method reduces the maximum APE from 6.5432 m to 3.4778 m, corresponding to a reduction of approximately 46.85%. These results demonstrate that the proposed semantic factor can improve the robustness of multi-source fusion localization in GNSS-degraded environments.

## 1. Introduction

Navigation and positioning provide the fundamental basis for environmental perception and planning control in autonomous driving and robotics. Obtaining accurate, continuous, reliable positioning and attitude information has become an urgent demand in related industries. Integrated navigation systems composed of the Global Navigation Satellite System (GNSS) and the Inertial Navigation System (INS) have been widely used because of their high accuracy and robustness. Since GNSS signals severely attenuate to an extremely weak power level upon reaching the ground, dense buildings and physical blockages in complex urban environments (e.g., urban canyons, elevated roads, and tunnels) cause a sharp drop in the carrier-to-noise ratio at the receiver tracking loops. This rapid degradation may lead to a loss of lock in the tracking loops, subsequently resulting in the degradation of the GNSS positioning performance. In this case, the GNSS/INS system may degrade into a pure inertial navigation mode, leading to rapid accumulation of positioning errors [1]. To ensure continuous positioning of the system, researchers have commonly introduced Light Detection and Ranging (LiDAR) and visual sensors for multi-source information fusion. Cameras and LiDAR are the most important sensors in Simultaneous Localization and Mapping (SLAM). In early studies, Smith et al. formulated localization and mapping as a unified state estimation problem and implemented pose estimation based on Kalman filtering. Later, Zhang et al. proposed the Lidar Odometry and Mapping(LOAM) algorithm, which estimates LiDAR odometry by extracting edge and planar features [2]. However, this method lacks a global optimization mechanism and tends to produce accumulated drift in large-scale scenarios. Shan et al. proposed Lightweight and Ground-Optimized Lidar Odometry and Mapping (LeGO-LOAM) [3], which improves algorithm performance through ground segmentation and loop-closure detection. In LiDAR-inertial fusion, the Fast LiDAR-Inertial Odometry (FAST-LIO) series achieves tightly coupled LiDAR-inertial optimization based on an iterated error-state Kalman filter, showing good performance in both accuracy and real-time capability [4,5]. Furthermore, LiDAR-Visual-Inertial Smoothing and Mapping (LVI-SAM) and Robust, Real-time, LiDAR-Inertial-Visual state Estimator (R2LIVE) integrate LiDAR, visual, and inertial information, achieving higher-accuracy localization and mapping.

With the development of related research, Factor Graph Optimization (FGO) has gradually become an important technical route for multi-source fusion. A factor graph can transform the state estimation problem into a sparse nonlinear optimization problem and realize unified modeling by introducing multiple sensor constraints. Kaess et al. proposed incremental Smoothing and Mapping 2(iSAM2), which significantly improves optimization efficiency through an incremental update mechanism [6]. Lidar Inertial Odometry via Smoothing and Mapping (LIO-SAM) incorporates IMU preintegration and LiDAR constraints into a factor graph framework, achieving high-precision real-time localization [7]. However, most of the above methods rely on geometric features such as point clouds, corners, and edges. In environments such as long corridors, open areas, or repetitive structures, insufficient geometric features may lead to degradation of system constraints and further affect positioning accuracy.

To overcome the limitations of geometric features, researchers have recently begun to introduce semantic information to assist SLAM. Deep-learning-based object detection methods, such as You Only Look Once (YOLO), can extract semantic categories and location information from images and have been used for dynamic object removal or semantic constraint construction. For example, DS-SLAM and DynaSLAM remove dynamic features through semantic segmentation, improving system robustness in dynamic environments [8,9]. CubeSLAM and QuadricSLAM model semantic objects as three-dimensional landmarks, realizing object-level SLAM [10,11]. In addition, navigation methods based on pre-built semantic landmarks have also received increasing attention. By matching fixed structural semantic information, localization accuracy can be effectively improved [12,13]. Nevertheless, although semantic information has been applied in multi-source fusion systems, most existing studies still focus on dynamic feature removal or local mapping. How to use semantic information to provide stable global constraints remains a problem that lacks a unified and effective solution.

In summary, existing multi-source integrated positioning methods still face the following problems in complex environments. First, under GNSS-degraded conditions, effective global constraints are absent, and system errors tend to accumulate continuously. Second, LiDAR and visual observations rely on geometric features. In degraded scenarios, the performance of LiDAR and visual odometry may become unstable. Third, semantic information has not been sufficiently incorporated into factor graph optimization to provide stable global constraints. To address these issues, this paper proposes a multi-source integrated positioning method incorporating semantic information based on a factor graph optimization framework. Fixed traffic lights are selected as semantic landmarks. A YOLOv8 object detection network is used to extract the pixel coordinates of the detection-box center and the corresponding confidence score. Then, depth information, camera pose, and the prior global coordinates of semantic landmarks are combined to establish a three-dimensional semantic target inversion model, which transforms two-dimensional image information into three-dimensional position estimates in the world coordinate system. Semantic factors are then constructed and incorporated into backend optimization, allowing them to work jointly with the Inertial Measurement Unit (IMU) preintegration factors and other observation factors. In addition, pixel localization error, depth estimation error, camera pose error, and landmark prior coordinate error are analyzed, and a noise covariance model for semantic factors is established. This model improves the stability of semantic constraints. Experimental results show that, under GNSS-degraded conditions, the introduction of semantic factors can provide effective global constraints, suppress error accumulation, and improve the accuracy and robustness of the multi-source integrated positioning system. The main contributions of this paper are summarized as follows:

1. A 2D-to-3D inversion model based on fixed semantic landmarks is proposed. Fixed traffic lights are selected as sparse semantic landmarks in urban environments. By combining the bounding-box center extracted by object detection, depth information, camera pose, and the prior global coordinates of semantic landmarks, two-dimensional image observations are transformed into three-dimensional position constraints in the world coordinate system.
2. A noise covariance matrix for the semantic factor is derived. The uncertainty sources of semantic observations, including pixel localization error, depth estimation error, camera pose error, and landmark prior coordinate error, are analyzed. Their propagation to the semantic residual is further modeled, enabling the semantic factor to be weighted according to observation uncertainty in backend factor graph optimization.
3. An unmanned ground vehicle experimental platform is built for validation. Through trajectory comparison, position error, attitude error, and statistical evaluation, the ability of the factor quality control method to suppress abnormal observations is verified, and the effect of semantic information fusion on positioning accuracy and robustness under GNSS-degraded conditions is analyzed.

## 2. Tightly Coupled Integrated Navigation Framework Based on Factor Graph Optimization

**Figure 1:**
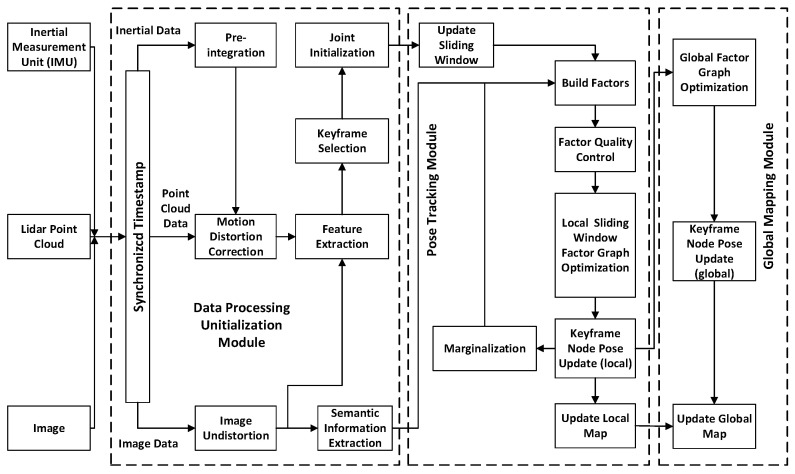
System block diagram.

To achieve accurate and robust navigation in complex environments, a tightly coupled multi-source integrated navigation framework based on factor graph optimization is constructed in this paper. The system framework is shown in Figure 1. The proposed framework takes IMU as the core sensor and fuses Global Navigation Satellite System (GNSS), LiDAR, and visual information in a unified optimization framework. It mainly consists of a data processing and system initialization module, a state prediction module, a pose update module, and a global mapping module.

In the data processing and system initialization module, multi-source measurements from the IMU, LiDAR, and camera are synchronized in time and unified in coordinate frames. The initial attitude, velocity, and sensor bias states are then estimated. IMU measurements are continuously obtained at a high frequency and provide short-term motion information for the system. LiDAR and visual sensors provide relative pose observations, which are used to construct constraints between adjacent keyframes in the backend optimization. GNSS measurements provide absolute position constraints when available. Through IMU preintegration, high-frequency inertial measurements are compressed into relative motion constraints between keyframes. The preintegrated result also provides a prior estimate for the current state, which is used as the initial value for backend optimization.

In the pose update module, IMU preintegration factors, LiDAR odometry factors, visual odometry factors, and GNSS factors are jointly modeled in a factor graph. The system state is updated by nonlinear optimization within a sliding window. To improve robustness, a factor quality- control mechanism is introduced to detect and process abnormal observation factors, thereby reducing the influence of low-quality measurements on state estimation. In addition, marginalization is used to maintain a bounded computational cost. In the global mapping module, loop-closure constraints are constructed by loop detection. A global factor graph is then optimized to further reduce accumulated errors and obtain globally consistent poses and maps.

In the multi-source integrated navigation system, measurements from different sensors are modeled as factors in the graph, and the system states are represented as nodes. The state of the vehicle is jointly estimated by minimizing the residuals of all measurement factors. Assuming that the sliding window contains N keyframes, the state variable set is defined as

(1) $$X=\left[x_{1},\cdots ,x_{k},\cdots ,x_{N}\right],$$

The state corresponding to the k th keyframe is defined as

(2) $$x_{k}={\left[p_{k}^{w},\;q_{k}^{w},\;v_{k}^{w},\;b_{a_{k}},\;b_{g_{k}}\right]}^{T},$$

where $p_{k}^{w}$, $q_{k}^{w}$, and $v_{k}^{w}$ denote the position, attitude, and velocity of the vehicle in the world frame, respectively. $b_{a_{k}}$ and $b_{g_{k}}$ are the accelerometer bias and gyroscope bias.

### 2.1. IMU Preintegration Residual

Considering the effects of bias, random noise, and environmental disturbances, the IMU measurement model is written as:

(3) $$\begin{array}{c} {\tilde{\omega }}_{ib}^{b}=\omega_{ib,}^{b}+b_{g}+w_{g}\\ {\tilde{f}}^{b}=f^{b}+b_{a}+w_{a} \end{array},$$

where ${\tilde{\omega }}_{ib}^{b}$ and ${\tilde{f}}^{b}$ denote the measured angular velocity and acceleration in the body frame, respectively. $\omega_{ib}^{b}$ and $f^{b}$ are the corresponding ideal values. $b_{g}$ and $w_{g}$ denote the gyroscope bias and measurement noise, while $b_{a}$ and $w_{a}$ denote the accelerometer bias and measurement noise, respectively. The superscripts b and w represent the body frame and the world frame.

According to [14], to avoid repeated integration caused by state iterations during optimization, IMU measurements between two consecutive keyframes are preintegrated. The IMU preintegration residual can be written as

(4) $$r_{imu}\left({\tilde{z}}_{k-1,k}^{Pre},X\right)=\left[\begin{array}{c} {\left(R_{b_{k-1}}^{w}\right)}^{T}\left(p_{b_{k}}^{w}-p_{b_{k-1}}^{w}-v_{b_{k-1}}^{w}\Delta t-\frac{1}{2}g^{w}\Delta t^{2}+\Delta p_{g/cor,k-1,k}^{w}\right)-{\hat{\alpha }}_{b_{k}}^{b_{k-1}}\\ {\left(R_{b_{k-1}}^{w}\right)}^{T}\left(v_{b_{k}}^{w}-v_{b_{k-1}}^{w}-g^{w}\Delta t+\Delta v_{g/cor,k-1,k}^{w}\right)-{\hat{\beta }}_{b_{k}}^{b_{k-1}}\\ 2{\left[{\left(q_{b_{k-1}}^{w}\right)}^{-1}⊗q_{b_{k}}^{w}⊗{\left({\hat{\gamma }}_{b_{k}}^{b_{k-1}}\right)}^{-1}\right]}_{xyz}\\ b_{g_{k}}-b_{g_{k-1}}\\ b_{a_{k}}-b_{a_{k-1}} \end{array}\right],$$

where Δt is the time interval between two frames. $p_{b}^{w}$, $R_{b}^{w}$, $q_{b}^{w}$ and $v_{b}^{w}$ denote the position, rotation matrix, attitude quaternion, and velocity of the body frame with respect to the world frame, respectively. $R_{b_{k-1}}^{w}$ denotes the rotation matrix from the body frame at time $t_{k-1}$ to the world frame. $g^{w}$ is the gravity vector in the world frame. The terms ${\hat{\alpha }}_{b_{k}}^{b_{k-1}}$, ${\hat{\beta }}_{b_{k}}^{b_{k-1}}$, ${\hat{\gamma }}_{b_{k}}^{b_{k-1}}$ denote the preintegrated position, velocity, and attitude measurements from time $t_{k-1}$ to $t_{k}$, respectively. $\Delta v_{g/cor,k-1,k}^{w}$ and $\Delta p_{g/cor,k-1,k}^{w}$ are the velocity and position correction terms caused by gravity and Coriolis acceleration [14]. ⊗ denotes quaternion multiplication, and ${\left[\cdot \right]}_{xyz}$ extracts the three-dimensional vector part of a quaternion.

### 2.2. GNSS Residual

When valid satellite signals are available, the GNSS receiver provides absolute global constraints. By transforming the raw GNSS measurements from the navigation frame n to the world frame w, we obtain the global position and velocity observations, denoted as ${\hat{p}}_{GNSS,i}^{w}$ and ${\hat{v}}_{GNSS,i}^{w}$ at the i-th epoch. To account for the spatial offset between the sensors, the GNSS residual is directly formulated as

(5) $$r_{GNSS}\left({\hat{z}}_{i}^{GNSS},X\right)=\left[\begin{array}{c} v_{i}^{w}+R_{b_{i}}^{w}\left(\left[\omega_{ib}^{b}\times \right]l_{GNSS}^{b}\right)-{\hat{v}}_{GNSS,i}^{w}\\ p_{i}^{w}+R_{b_{i}}^{w}l_{GNSS}^{b}-{\hat{p}}_{GNSS,i}^{w} \end{array}\right],$$

where $p_{i}^{w}$, $v_{i}^{w}$, and $R_{b_{i}}^{w}$ denote the position, velocity, and rotation matrix of the IMU body frame in the world frame at epoch i, respectively. $\omega_{ib}^{b}$ denotes the angular velocity expressed in the body frame, ${\left[\cdot \right]}_{\times }$ denotes the skew-symmetric matrix of a vector, and $l_{GNSS}^{b}$ denotes the lever arm of the GNSS antenna.

### 2.3. LIO Residual

The LiDAR SLAM subsystem estimates the vehicle pose using the three-dimensional point cloud acquired by the LiDAR. Its core idea is to estimate the motion of the vehicle by establishing geometric constraints between adjacent point cloud frames. In the front-end processing stage, features are first extracted from the raw point cloud. By analyzing the local curvature of the point cloud, points are divided into edge feature points and planar feature points. Edge features are used to describe local geometric discontinuities, while planar features are used to describe locally smooth structures. The extracted features are then used to establish correspondences between the current frame and historical frames.

In the pose estimation stage, LiDAR SLAM constructs geometric constraints through feature matching. For edge features, the point-to-line distance is usually used as the error term. For planar features, the point-to-plane distance is used to construct the residual. The relative pose between adjacent frames is obtained by minimizing these geometric residuals. This process is usually formulated as a nonlinear least-squares optimization problem and solved iteratively. The relative pose between adjacent keyframes is computed as

(6) $$\Delta {\tilde{T}}_{k-1,k}^{lio}=\arg\min_{T_{k-1}^{k}}\left\{\sum f_{e}\left(T_{k-1}^{k}\right)+\sum f_{p}\left(T_{k-1}^{k}\right)\right\},$$

where $\Delta {\tilde{T}}_{k-1,k}^{lio}$ denotes the estimated relative pose from the k−1 frame to the  k frame. $T_{k-1}^{k}$ is the pose transformation to be optimized. $f_{e}\left(\cdot \right)$ and $f_{p}$ denote the residual functions corresponding to edge and planar features.

The relative pose output by the LIO subsystem is used as the backend observation. The observation is written as

(7) $${\tilde{z}}_{k-1,k}^{lio}=\Delta {\tilde{T}}_{k-1,k}^{lio},$$

Therefore, the LIO residual is defined as

(8) $$r_{lio}\left({\tilde{z}}_{k-1,k}^{lio},X\right)=\mathrm{Log}{\left({\left(\Delta {\tilde{T}}_{k-1,k}^{lio}\right)}^{-1}T_{k-1}^{-1}T_{k}\right)}^{\vee },$$

$T_{k-1}$ and $T_{k}$ denote the poses of keyframes k − 1 and k in the backend optimization. Log(⋅) maps an element from the Lie group SE(3) to its Lie algebra and ${\left(\cdot \right)}^{\vee }$ denotes the vectorization operator that maps a Lie algebra matrix to a vector.

### 2.4. VIO Residual

The visual SLAM subsystem estimates the vehicle pose using image information acquired by the camera. By extracting image features and establishing geometric constraints between multiple frames, the continuous pose of the vehicle can be estimated. In this paper, ORB features are adopted in the front-end processing. ORB combines FAST corner detection and BRIEF descriptors, which provide good computational efficiency as well as rotation and scale invariance [15]. The extracted feature points are matched between adjacent frames to establish image correspondences.

In the pose estimation stage, visual SLAM uses multi-view geometry to build correspondences between feature points in different image frames. The camera pose is estimated by constructing reprojection error constraints. In a sliding window, all observed feature points are jointly optimized, and the keyframe poses are obtained by nonlinear least-squares optimization [16]. The relative pose between adjacent keyframes is then used as the visual odometry measurement and passed to the backend optimization. The observation is written as

(9) $${\tilde{z}}_{k-1,k}^{vio}=\Delta {\tilde{T}}_{k-1,k}^{vio},$$

Thus, the VIO residual is defined as

(10) $$r_{vio}\left({\tilde{z}}_{k-1,k}^{vio},X\right)=\mathrm{Log}{\left({\left(\Delta {\tilde{T}}_{k-1,k}^{vio}\right)}^{-1}T_{k-1}^{-1}T_{k}\right)}^{\vee },$$

### 2.5. Prior Residual

Marginalization is used to compress the constraint information associated with the eliminated states, and the remaining information is retained as a prior factor for subsequent optimization. The prior residual is constructed as

(11) $$r_{p}(X)=b_{p}-H_{p}X,$$

where $b_{p}$ and $H_{p}$ are obtained from the Schur complement [17] during marginalization.

### 2.6. Backend Optimization Function

Based on the state definition above, the state variable set X, defined in Equation (1), is estimated by minimizing the residuals of the IMU, GNSS, LiDAR odometry, and visual odometry factors. The backend optimization objective is written as

(12) $$X^{*}=\arg\min_{X}\left\{\begin{array}{l} {\left\|r_{p}(X)\right\|}^{2}+\sum_{k=1}^{n}{\left\|r_{imu}\left({\tilde{z}}_{k-1,k}^{Pre},X\right)\right\|}_{\Sigma_{imu}^{k}}^{2}+\sum_{k=1}^{n}{\left\|r_{gnss}\left({\tilde{z}}_{k}^{GNSS},X\right)\right\|}_{\Sigma_{gnss}^{k}}^{2}\\ +\sum_{k=1}^{n}{\left\|r_{lio}\left({\tilde{z}}_{k-1,k}^{lio},X\right)\right\|}_{\Sigma_{lio}^{k}}^{2}+\sum_{k=1}^{n}{\left\|r_{vio}\left({\tilde{z}}_{k-1,k}^{vio},X\right)\right\|}_{\Sigma_{vio}^{k}}^{2} \end{array}\right\},$$

In this objective function, each residual term represents the constraint imposed by a specific sensor observation on the system state. Σ denotes the uncertainty weight of the corresponding observation. The terms $r_{p}(X)$, $r_{imu}\left({\tilde{z}}_{k-1,k}^{Pre},X\right)$, $r_{gnss}\left({\tilde{z}}_{k}^{GNSS},X\right)$, $r_{lio}\left({\tilde{z}}_{k-1,k}^{lio},X\right)$, $r_{vio}\left({\tilde{z}}_{k-1,k}^{vio},X\right)$ denote the prior residual, IMU preintegration residual, GNSS position residual, LiDAR odometry residual, and visual odometry residual, respectively.

### 2.7. Factor Quality Control Strategy

To reduce the influence of low-quality measurements on backend optimization, a factor quality control strategy is introduced in the proposed multi-source fusion framework. In complex urban environments, GNSS measurements may be affected by signal blockage and multipath propagation, LiDAR odometry may degrade in geometrically degenerated scenarios, and visual odometry may become unreliable under illumination changes, weak textures, or feature-tracking failures. If these low-quality observations are directly inserted into the factor graph without checking, erroneous constraints may be introduced and may degrade the state estimation accuracy.

In this paper, IMU preintegration is used as a local motion reference because IMU measurements have a high frequency and can provide short-term motion prediction after bias correction. For two adjacent keyframes k-1 and k, the IMU preintegration provides a relative motion prediction $\Delta T_{k-1,k}^{imu}$, while the LIO and VIO subsystems provide relative pose observations $\Delta T_{k-1,k}^{lio}$ and $\Delta T_{k-1,k}^{vio}$, respectively. The consistency residual is defined as

(13) $$r_{check}^{s}=\mathrm{Log}{\left[{\left(\Delta T_{k-1,k}^{imu}\right)}^{-1}\Delta T_{k-1,k}^{s}\right]}^{\vee },\;\;\;s\in \{\mathrm{lio},\mathrm{vio}\},$$

where Log(⋅) maps an element from $SE\left(3\right)$ to its Lie algebra, and ${\left(\cdot \right)}^{\vee }$ denotes the vectorization operator. A smaller consistency residual indicates that the corresponding external odometry observation is more consistent with the local motion predicted by IMU preintegration. For simplicity, the consistency residual of either the LIO or VIO subsystem is denoted as $r_{k}$, and its covariance matrix is denoted as $\Sigma_{k}$. The Mahalanobis distance of the residual is used as the chi-square test statistic:

(14) $$\gamma_{k}=r_{k}^{T}\Sigma_{k}^{-1}r_{k},$$

Under normal conditions, $\gamma_{k}$ approximately follows a chi-square distribution with m degrees of freedom, where m=6 for the relative pose residual. Given the significance level α, the threshold is defined as

(15) $$T_{\alpha }=\chi_{m,\alpha }^{2},$$

The single-frame chi-square test can detect abrupt large faults, but its sensitivity to small or slowly varying degradation is limited. To improve the detection capability for gradual degradation, a sliding-window statistic is further introduced. For the current time k, a residual window with length N is defined as

(16) $$\left\{r_{k-N+1},r_{k-N+2},\cdots ,r_{k}\right\},$$

The weighted mean residual in the window is calculated as

(17) $$\begin{array}{l} {\bar{r}}_{k}=W_{k}\sum_{i=k-N+1}^{k}\Sigma_{i}^{-1}r_{i}\\ W_{k}^{-1}=\sum_{i=k-N+1}^{k}\Sigma_{i}^{-1}, \end{array}$$

Then, the sliding-window test statistic is defined as

(18) $$\Gamma_{k}={\bar{r}}_{k}^{T}W_{k}^{-1}{\bar{r}}_{k},$$

Under normal conditions, $\Gamma_{k}$ approximately follows a chi-square distribution with m degrees of freedom. When continuous degradation or a slowly varying fault occurs, the statistic tends to increase because the residual deviations are accumulated in the window. Therefore, the sliding-window statistic is more sensitive to gradual degradation than the single-frame statistic.

In this paper, a dual-threshold robust weighting strategy is adopted. $T_{1}$ and $T_{2}$ denote two chi-square upper-tail thresholds satisfying $T_{1}<T_{2}$. The robust equivalent weight is defined as

(19) $$w_{k}=\left\{\begin{array}{ll} 1, & \Gamma_{k}\leq T_{1},\\ \frac{T_{2}-\Gamma_{k}}{T_{2}-T_{1}}, & T_{1}<\Gamma_{k}<T_{2},\\ 0, & \Gamma_{k}\geq T_{2}. \end{array}\right.,$$

When $\Gamma_{k}$ is below the lower threshold $T_{1}$, the corresponding factor is regarded as reliable and is assigned full weight. When $\Gamma_{k}$ exceeds the upper threshold $T_{2}$, the factor is considered severely abnormal and is rejected. When $\Gamma_{k}$ lies between the two thresholds, the factor is regarded as degraded, and its influence on the backend optimization is smoothly reduced through linear down-weighting. After the equivalent weight is obtained, the covariance matrix of the corresponding factor is adjusted as

(20) $$\Sigma_{k}^{*}=\frac{1}{w_{k}}\Sigma_{k},$$

Thus, a smaller weight leads to a larger covariance and a smaller information contribution in the backend factor graph optimization. When $w_{k}=0$, the corresponding factor is directly rejected and is not inserted into the factor graph to avoid numerical singularity.

This quality control strategy is mainly applied to the LIO and VIO relative-pose factors by checking their consistency with IMU preintegration. For GNSS factors, the positioning status and quality flag are checked before factor construction, and invalid or unavailable GNSS measurements are not inserted into the factor graph. For semantic factors, detections with low confidence, invalid depth values, or failed landmark association are rejected before semantic factor construction. Therefore, the proposed strategy reduces the influence of low-quality measurements while preserving reliable multi-source constraints in the backend factor graph.

## 3. Multi-Source Integrated Navigation Algorithm Incorporating Semantic Information

Traditional integrated navigation and multi-sensor fusion localization methods mainly rely on geometric features, texture features, and inertial motion information for state estimation. In complex urban environments, GNSS signals are easily affected by building blockage, multipath effects, and other factors. As a result, GNSS/INS/LiDAR/visual integrated navigation systems may lose global positioning constraints, and accumulated errors may continue to increase. To address this problem, semantic factors are introduced in this section to provide additional global constraints for the integrated navigation system.

### 3.1. YOLO-Based Semantic Object Detection

The key to obtaining semantic information lies in accurate and efficient object recognition and localization in the scene. To introduce high-level semantic information into the multi-source integrated navigation system, a deep-learning-based object detection method is adopted to identify fixed semantic landmarks in the environment. Compared with conventional geometric and texture features, semantic landmarks usually correspond to physical objects with clear meanings, such as traffic signs, road signs, or fixed infrastructure. These objects can provide stable absolute reference constraints for the system.

In this paper, YOLOv8 is adopted as the semantic object detection model. The YOLO series belongs to one-stage object detection methods. Its main idea is to transform the object detection task into an end-to-end regression problem. The object position and category probability can be directly predicted from the input image. YOLOv8 [18] is an updated version released by Ultralytics after YOLOv5. It supports multiple visual tasks, including image classification, object detection, and instance segmentation. Considering the representativeness and practical significance of traffic signal lights in road scenarios, fixed traffic signal lights are selected as semantic landmarks in this work, and YOLOv8 is used for model training and object detection.

**Figure 2:**
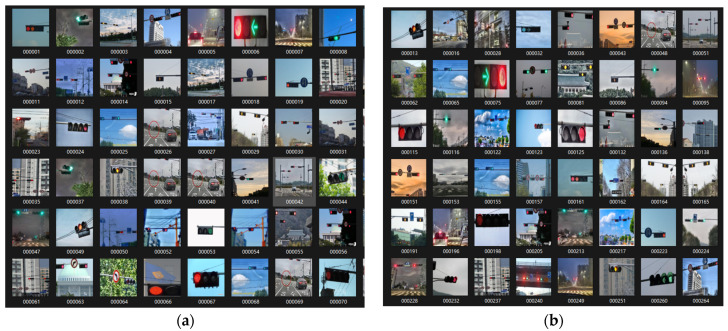
Sample thumbnails of the traffic light dataset. (**a**) Training set; (**b**) Validation set.

During dataset construction, traffic signal images were collected under different time periods, weather conditions, and viewing angles. The dataset was then manually annotated. A total of 423 training images and 108 validation images were used. The original image resolution was 640 × 360 pixels. The annotation includes the overall traffic signal target box and the specific signal categories. The constructed dataset covers different signal states, including red, yellow, and green lights, and also includes varying distances, orientations, object scales, and partial occlusion cases. Therefore, it can reasonably reflect the observation characteristics of traffic signal lights in real road scenarios. As shown in Figure 2, representative sample thumbnails from the training and validation sets are presented to illustrate the diversity of the constructed traffic light dataset.

During model training, the targets in each image are manually annotated. The annotations mainly include the bounding-box position and category information of traffic signal lights, and the corresponding label files are generated for all images.

After training, the YOLOv8 model can accurately detect traffic signal targets in road scenes and output the corresponding class labels and detection confidences. Under different illumination conditions, different observation distances, and scenes with multiple targets, the trained model maintains stable detection performance and can output complete detection positions of traffic signal lights. This indicates that the dataset is representative of real road scenarios and that the trained model can effectively support the semantic detection of traffic signal lights. It also provides reliable front-end perception information for the subsequent extraction of target center pixel coordinates and semantic three-dimensional position inversion.

**Figure 3:**
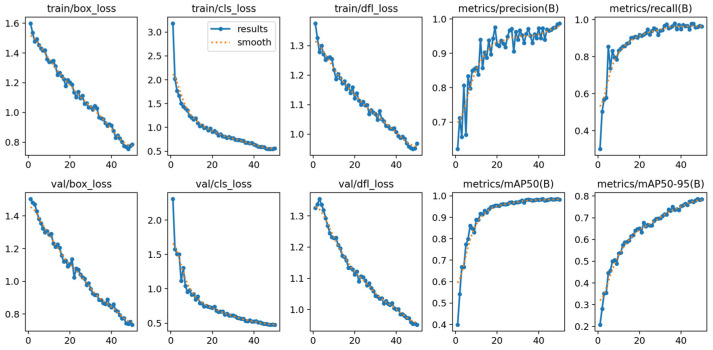
Training and validation curves of the YOLOv8n traffic-light detector.

The training and validation curves of the YOLOv8n traffic-light detector are shown in Figure 3. As the training epoch increases, the training losses, including box loss, classification loss, and DFL loss, decrease steadily. The validation losses also show a consistent downward trend, indicating that the detector does not suffer from obvious overfitting. Meanwhile, the precision, recall, mAP@0.5, and mAP@0.5:0.95 increase rapidly in the early training stage and gradually converge in the later stage. At the end of training, the precision and recall are both close to 0.98, the mAP@0.5 is close to 0.98, and the mAP@0.5:0.95 is approximately 0.78. These results indicate that the trained YOLOv8n model can provide reliable traffic-light detection results for subsequent semantic factor construction.

**Figure 4:**
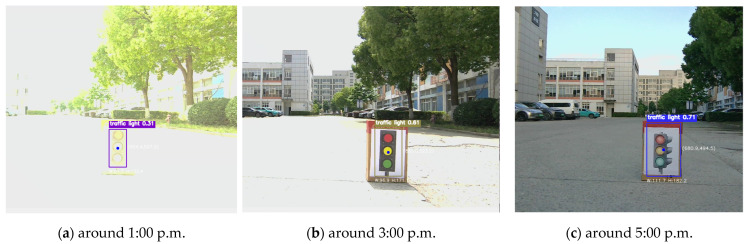
Detection results of the YOLOv8n traffic-light detector under different illumination conditions.

The influence of illumination conditions on YOLOv8n detection is shown in Figure 4. The three images were collected at approximately 1:00 p.m., 3:00 p.m., and 5:00 p.m., respectively. It can be observed that the traffic-light landmark can be detected under all three illumination conditions, but the detection confidence varies with the illumination intensity. Around 1:00 p.m., the scene is affected by strong sunlight and local overexposure, which reduces the image contrast of the traffic light and leads to a relatively low confidence score of 0.51. Around 3:00 p.m., the overexposure effect is weakened, and the traffic-light region becomes clearer, increasing the confidence score to 0.61. Around 5:00 p.m., the illumination becomes more stable, and the target boundary is more distinguishable, resulting in a higher confidence score of 0.70. These results indicate that the trained YOLOv8n detector can still detect traffic-light landmarks under different illumination conditions, but strong illumination and local overexposure may reduce detection confidence. Since semantic factors provide absolute constraints in the backend factor graph, low-confidence detections should not be directly used to construct semantic factors. Therefore, in this paper, only detections with confidence scores higher than 0.5 are accepted for semantic factor construction. Detections lower than this threshold are rejected before being inserted into the factor graph. For accepted detections, the confidence score is further used to adjust the semantic factor covariance, so that observations with lower confidence are assigned larger covariance and smaller weight in the backend optimization. This strategy improves the robustness of semantic factor construction under illumination variations.

**Figure 5:**
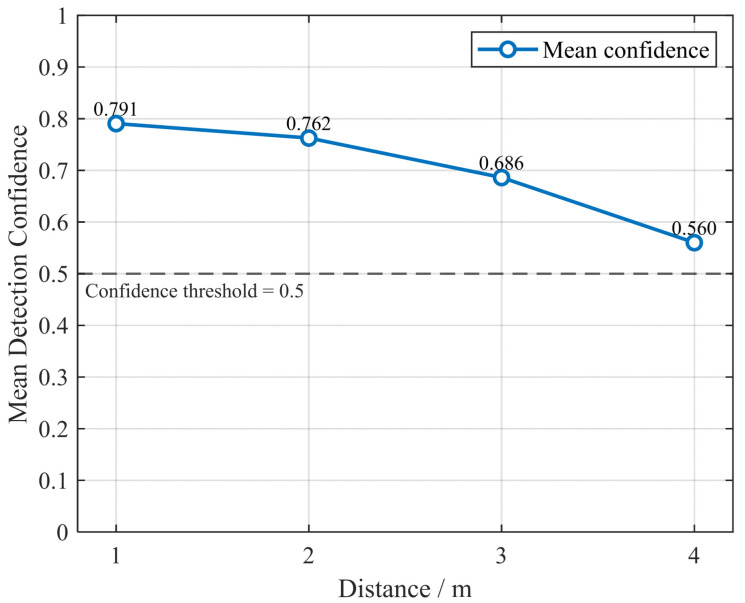
Influence of landmark distance on YOLOv8n detection confidence.

To analyze the influence of observation distance on the YOLOv8n detection confidence, the camera was placed at approximately 1 m, 2 m, 3 m, and 4 m from the traffic-light landmark. At each distance, the camera was kept stationary for 10 s, and two confidence values were collected per second. The mean confidence at each distance was then calculated, as shown in Figure 5. It can be observed that the detection confidence decreases as the observation distance increases. The mean confidence values are 0.791, 0.762, 0.686, and 0.560 at 1 m, 2 m, 3 m, and 4 m, respectively. This is because the apparent size of the traffic light in the image decreases with increasing distance, making the target more susceptible to image noise, background interference, and localization uncertainty.

The mean confidence at 4 m is still higher than the confidence threshold of 0.5. This indicates that the trained YOLOv8n detector can still provide valid traffic-light observations within the tested distance range. In the proposed framework, only detections with confidence scores higher than 0.5 are used for semantic factor construction. Therefore, low-confidence detections are rejected before being inserted into the backend factor graph. In addition, for accepted detections, the confidence score is further used to adjust the semantic factor covariance, so that observations with lower confidence contribute smaller weights in the backend optimization. This mechanism reduces the risk of introducing unreliable semantic constraints caused by long-distance observations.

**Figure 6:**
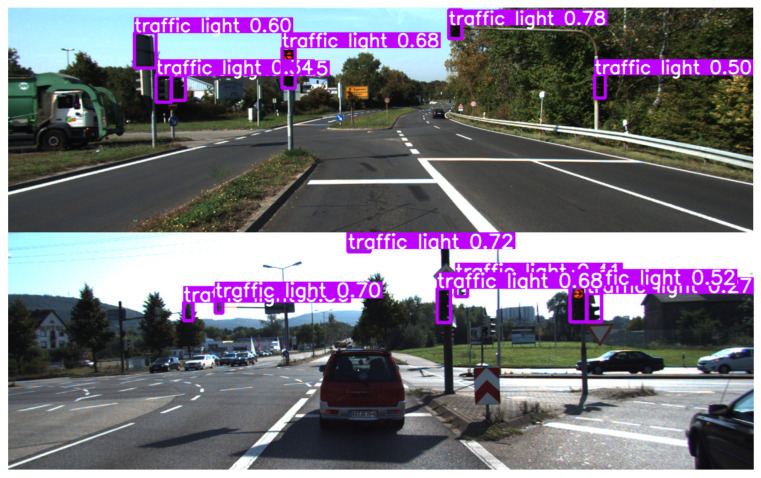
Traffic-light detection examples on the KITTI 00 sequence.

Next, the generalization ability of the YOLOv8n detector in previously unexplored environments was tested on the KITTI 00 sequence. Compared with the self-collected dataset, the KITTI 00 sequence contains more complex real-world road scenarios, including different road layouts, illumination changes, traffic flow, and background conditions. A total of 7480 images were tested, and 2808 traffic-light targets were detected. Several representative detection examples are shown in Figure 6, indicating that the detector can still identify traffic-light targets in unfamiliar urban road scenes.

**Figure 7:**
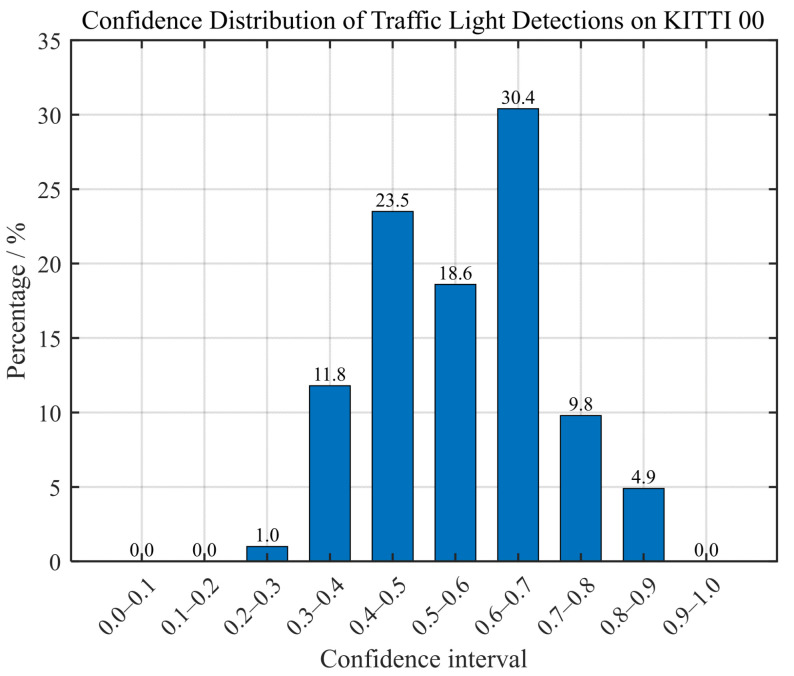
Confidence distribution of traffic-light detections on the KITTI 00 sequence.

Figure 7 also presents the confidence distribution of the traffic-light detections. The results show that most detections are concentrated in the confidence range of 0.4–0.8. Specifically, 18.6% of the detections fall within 0.5–0.6, 30.4% within 0.6–0.7, and 9.8% within 0.7–0.8. The proportions of detections with confidence lower than 0.3 or higher than 0.9 are relatively small. These results indicate that the detector has a certain degree of generalization ability on the KITTI 00 sequence. They also show that the YOLOv8n detector can generalize to previously unseen urban road scenes to some extent, although its detection confidence is still affected by environmental changes.

The proposed semantic factor framework is not limited to this specific object type. The essential requirement of a semantic landmark is that it should be static, repeatedly observable, distinguishable by the perception module, and associated with a known global coordinate or map prior. Therefore, the same formulation can be extended to other fixed urban objects, such as traffic signs, road lines, building corners, road-side facilities.

For different semantic objects, the observation model and uncertainty model may be slightly different. For example, traffic signs and traffic lights can be represented by bounding-box center observations and depth measurements, while buildings or large structures may require more stable geometric features such as corners, edges, or planes. Once the object position or geometric feature can be transformed into the world frame, the semantic residual can still be constructed by comparing the estimated landmark position with its prior coordinate.

It should also be noted that the prior coordinates of semantic landmarks do not necessarily have to be manually measured one by one using RTK. In this paper, pre-measured traffic lights are used as a representative and controllable case to verify the feasibility of introducing sparse fixed semantic landmarks into the factor graph. In future work, the proposed framework will be extended to multiple types of semantic landmarks and combined with HD-map-based landmark management to improve its scalability in large-scale urban environments.

### 3.2. Optimization Method Assisted by Semantic Information

#### 3.2.1. Three-Dimensional Coordinate Inversion with Depth Information

After the semantic target is detected, the two-dimensional image observation must be further converted into a measurement that can be used in the backend optimization of the integrated navigation system. In this paper, fixed traffic signal lights are selected as semantic landmarks. For each fixed semantic landmark, its global coordinate in the world frame is measured in advance. First, YOLOv8 is used to detect the bounding-box center pixel coordinate of the semantic target in the image. Then, depth information and camera intrinsic parameters are combined to recover the three-dimensional position of the target in the current camera frame. By further using the extrinsic transformation between the camera and the vehicle body and the known global coordinate of the semantic target, the absolute position of the current vehicle in the world frame can be inferred. In this way, the semantic observation can be transformed into a global position measurement and introduced into the backend factor graph optimization as an additional constraint.

The pixel coordinate is mapped to the normalized image plane through the camera intrinsic parameters. The center pixel coordinate of the detected semantic target in the normalized image plane is expressed as:

(21) $${\tilde{p}}_{i}^{k}=K^{-1}\left[\begin{array}{c} u_{i}^{k}\\ v_{i}^{k}\\ 1 \end{array}\right],$$

where K is the camera intrinsic matrix, $u_{i}^{k}$ and $v_{i}^{k}$ denote the scalar horizontal and vertical pixel coordinates of the bounding-box center of the i-th semantic target in the k-th image frame, respectively. Combined with the target depth $d_{i}^{k}$ obtained from the depth camera, the three-dimensional position of the semantic target in the camera frame can be written as follows.

(22) $$P_{i}^{c_{k}}=d_{i}^{k}{\tilde{p}}_{i}^{k}=d_{i}^{k}K^{-1}\left[\begin{array}{c} u_{i}^{k}\\ v_{i}^{k}\\ 1 \end{array}\right],$$

Here, $P_{i}^{c_{k}}$ denotes the three-dimensional position of the semantic target in the current camera frame $c_{k}$. ${\tilde{p}}_{i}^{k}$ denotes the coordinates of the pixel point after back-projection onto the normalized image plane.

In practical implementation, the depth value of a semantic landmark is not directly taken from a single center pixel, because the bounding-box center may be affected by partial occlusion, viewing angle changes, or detection jitter. To improve robustness, a local depth window centered at the bounding-box center is used. Specifically, for the detected center pixel $\left(u_{i}^{k},v_{i}^{k}\right)$, a 10×10 pixel region around the center is selected from the depth image. Invalid depth values, including NaN values, zero values, and values outside the valid depth range, are first removed. The median value of the remaining valid depth pixels is then used as the depth $d_{i}^{k}$ of the semantic landmark.

To transform this relative observation into the world frame, the camera pose with respect to the world frame is introduced as

(23) $$T_{c_{k}}^{w}=\left[\begin{array}{cc} R_{c_{k}}^{w} & t_{c_{k}}^{w}\\ 0^{T} & 1 \end{array}\right],$$

where $R_{c_{k}}^{w}$ is the rotation matrix from the camera frame to the world frame, and $t_{c_{k}}^{w}$ is the position of the camera at time k in the world frame. The semantic target position estimated from the current observation can then be transformed into the world frame as

(24) $${\hat{P}}_{i}^{w}=R_{c_{k}}^{w}P_{i}^{c_{k}}+t_{c_{k}}^{w},$$

Substituting Equation (22) into Equation (24) gives

(25) $${\hat{P}}_{i}^{w}=R_{c_{k}}^{w}\left(d_{i}^{k}K^{-1}\left[\begin{array}{c} u_{i}^{k}\\ v_{i}^{k}\\ 1 \end{array}\right]\right)+t_{c_{k}}^{w},$$

which is obtained by combining the normalized pixel coordinate defined in Equation (21), the depth-based 3D point defined in Equation (22), and the camera pose defined in Equation (23).

In Equation (25), the semantic target position ${\hat{P}}_{i}^{w}$ in the world frame is estimated jointly from the semantic image observation, depth information, and the current camera pose. It can be seen that, once the target center pixel coordinate, the corresponding depth value, and the current camera pose are known, the two-dimensional semantic observation in the image can be converted into a three-dimensional position estimate in the world frame.

#### 3.2.2. Semantic Factor Construction

The semantic landmarks used in this paper are fixed traffic signal lights that are selected and arranged in advance. Their global coordinates are measured before the experiment. In the RTK(Real-Time Kinematic) integrated navigation system, the accurate global coordinates of the semantic landmarks are first obtained by static measurement when the positioning quality is reliable. The measured latitude, longitude, and altitude are then transformed into the world coordinate frame through coordinate conversion. In this way, a global semantic landmark prior is obtained. Since RTK can provide highly accurate positioning measurements in open environments, the obtained semantic landmark coordinates can be used as known global information in the backend optimization. After being measured, the global coordinate of each semantic landmark remains fixed during system operation.

To facilitate real-world deployment, the prior positions of fixed semantic landmarks are pre-loaded into the system memory. Each semantic landmark is formulated as a lightweight data point containing only a unique ID and its geodetic coordinates, namely latitude, longitude, and altitude. A 4-byte integer is used for the ID, and double-precision values are used for the latitude, longitude, and altitude; each landmark requires approximately 28 bytes of storage. Therefore, even for a city-scale application with 10,000 traffic lights, the total memory footprint is only about 0.28 MB. During system initialization, the stored geodetic coordinates can be transformed into the local East-North-Up (ENU) frame or the predefined world frame for backend factor graph optimization. Compared with dense high-definition maps or continuous semantic maps, the proposed method relies only on sparse fixed landmarks, which significantly reduces the storage burden while providing supplementary global constraints in GNSS-degraded environments.

Assume that the known coordinate of the i-th fixed semantic landmark in the world frame is $P_{i}^{w}$. During system operation, after the semantic target is detected by YOLOv8 in the current image frame, the current observation can be back-projected into the world frame using the target center pixel coordinate, depth information, and camera pose. A semantic residual is then constructed by comparing the estimated semantic target position ${\hat{P}}_{i}^{w}$ with its known prior coordinate $P_{i}^{w}$. The residual is defined as

(26) $$r_{sem}={\hat{P}}_{i}^{w}-P_{i}^{w},$$

Substituting Equation (25) into Equation (26), the semantic residual can be written as

(27) $$r_{sem}=R_{c_{k}}^{w}\left(d_{i}^{k}K^{-1}\left[\begin{array}{c} u_{i}^{k}\\ v_{i}^{k}\\ 1 \end{array}\right]\right)+t_{c_{k}}^{w}-P_{i}^{w},$$

The above formulation shows that the semantic observation is essentially used to infer the global position of a fixed semantic landmark. Specifically, the two-dimensional center pixel observation and depth information are first used to recover the three-dimensional position of the target in the current camera frame. This position is then transformed into the world frame using the camera pose. By comparing the estimated global position with the known prior position of the semantic landmark, an absolute semantic constraint is established. Therefore, the semantic factor can provide a global reference similar to a position measurement and can constrain the global pose of the navigation system. To incorporate the semantic observation into the backend factor graph optimization, its uncertainty must be properly modeled. Let the covariance matrix corresponding to the semantic observation at time k be denoted as $\Sigma_{k}^{sem}$. The cost term of the semantic factor is then expressed as

(28) $${\left\|r_{sem}\left(z_{k}^{sem},X\right)\right\|}_{\Sigma_{k}^{sem}}^{2}=r_{sem}^{T}\Sigma_{k}^{sem^{-1}}r_{sem},$$

In addition, the detection confidence is used to evaluate the reliability of the semantic observation. Let $s_{i}^{k}$ denote the detection confidence of the i-th semantic landmark in the k-th image frame, and let $s_{\min}$ denote the minimum confidence threshold. In the experiment, the confidence threshold was set to $s_{\min}=0.5$. A semantic observation is accepted only when

(29) $$s_{i}^{k}\geq s_{\min},$$

Detections with confidence scores lower than $s_{\min}$ are rejected before semantic factor construction. For accepted semantic observations, the confidence score is further used to adjust the covariance of the semantic factor. A lower confidence score indicates lower observation reliability and therefore corresponds to a larger covariance. The confidence-weighted covariance is defined as

(30) $$\Sigma_{k}^{sem,*}=\frac{1}{\max(s_{i}^{k},s_{\min})}\Sigma_{k}^{sem},$$

where $\Sigma_{k}^{sem}$ is the semantic covariance matrix derived from pixel localization error, depth estimation error, camera pose error, and landmark prior coordinate error. The cost term of the semantic factor is then expressed as

(31) $${\left\|r_{sem}\left(z_{k}^{sem},X\right)\right\|}_{\Sigma_{k}^{sem,*}}^{2}=r_{sem}^{T}{\left(\Sigma_{k}^{sem,*}\right)}^{-1}r_{sem},$$

where $z_{k}^{sem}$ denotes the semantic observation at time k, which mainly includes the target center pixel coordinate, the corresponding depth value, the detection confidence, and the prior global coordinate of the fixed semantic landmark. After the semantic observation is introduced, the global optimization objective of the system can be extended as follows, where X denotes the state variable set defined in Equation (1):

(32) $$X^{*}=\arg\min_{X}\left\{\begin{array}{l} \|r_{p}(X){\|}^{2}+\sum_{k=1}^{n}{\left\|r_{imu}(z_{k-1,k}^{pre},X)\right\|}_{\Sigma_{imu}}^{2}+\sum_{k=1}^{n}{\left\|r_{gnss}(z_{k}^{GNSS},X)\right\|}_{\Sigma_{gnss}}^{2}\\ +\sum_{k=1}^{n}{\left\|r_{lio}(z_{k-1,k}^{lio},X)\right\|}_{\Sigma_{k}^{lio}}^{2}+\sum_{k=1}^{n}{\left\|r_{vio}(z_{k-1,k}^{vio},X)\right\|}_{\Sigma_{k}^{vio}}^{2}+\sum_{k=1}^{n_{sem}}{\left\|r_{sem}(z_{k}^{sem},X)\right\|}_{\Sigma_{k}^{sem,*}}^{2} \end{array}\right\}$$

**Figure 8:**
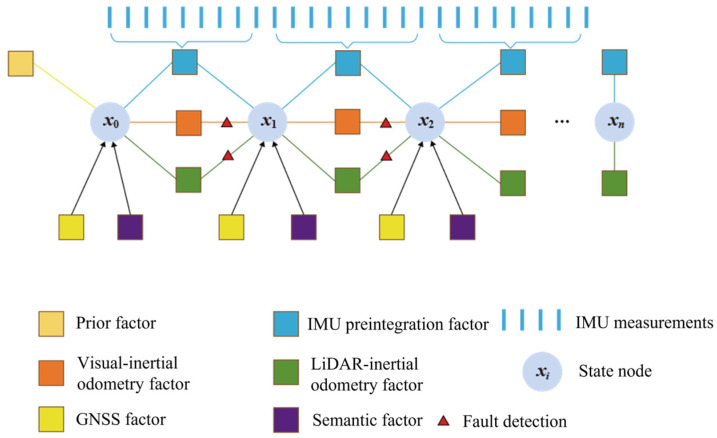
Schematic of the factor graph.

Here, $n_{sem}$ denotes the number of valid semantic observations involved in the sliding-window optimization. The semantic factor has the same residual-squared form as the other measurement factors, and can therefore be incorporated into the unified factor graph optimization framework. Different from the preceding factors, the semantic factor provides an absolute position-related constraint based on fixed semantic landmarks. It can therefore play a role similar to a global reference in the backend optimization. When GNSS is available, the semantic factor can serve as a supplementary absolute position measurement. When GNSS positioning is denied or GNSS is unavailable, the semantic factor can provide additional global position reference information and enhance the long-term positioning capability of the integrated navigation system. The factor graph structure is shown in Figure 8.

**Figure 9:**
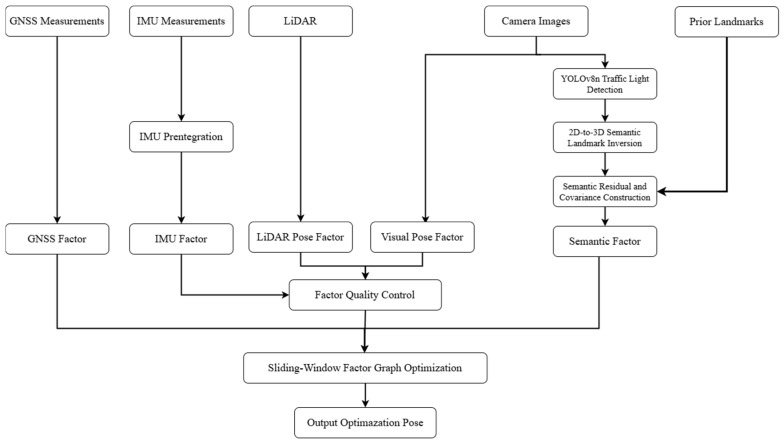
Flow chart of the factor graph.

To further clarify the overall implementation process of the proposed method, the system algorithm flowchart is shown in Figure 9.

#### 3.2.3. Noise Covariance Matrix of the Semantic Factor

According to Equation (27), the errors of the semantic observation mainly come from the following sources: the pixel localization error of the target center, the depth estimation error, the camera pose error, and the prior coordinate error of the fixed semantic landmark. In addition, the camera intrinsic calibration error, the deviation between the detected bounding-box center and the true projection center of the target, and the geometric approximation error introduced by the target depth can also be propagated into the final semantic measurement.

Based on the semantic residual defined in Equation (27), first-order perturbation is applied to the semantic observation model. The perturbation of the semantic residual can be expressed as

(33) $$\delta r_{sem}\approx J_{u}\delta u_{i}^{k}+J_{v}\delta v_{i}^{k}+J_{d}\delta d_{i}^{k}+J_{pose}\delta \xi_{c_{k}}^{w}+J_{lm}\delta P_{i}^{w},$$

where $J_{u}$, $J_{v}$, $J_{d}$, $J_{pose}$, $J_{lm}$ are the Jacobian matrices of the semantic residual with respect to the horizontal pixel coordinate, vertical pixel coordinate, depth value, camera pose, and prior global coordinate of the fixed semantic landmark, respectively. $\delta u_{i}^{k}$ and $\delta v_{i}^{k}$ denote the perturbations of the detected center of the i-th semantic target in the k-th image frame along the horizontal and vertical pixel coordinates, respectively. $\delta d_{i}^{k}$ denotes the perturbation of the depth measurement of the semantic target. $\delta \xi_{c_{k}}^{w}$ denotes the small perturbation of the camera pose at frame k in the Lie algebra space, which is usually composed of translational and rotational perturbations. $\delta P_{i}^{w}$ denotes the perturbation of the prior global coordinate of the fixed semantic landmark.

(1) Pixel observation noise modeling:

The geometric center of the detection bounding box $\left(u_{i}^{k},v_{i}^{k}\right)$ is taken as the pixel observation of the semantic target. Its measurement model is written as:

(34) $$\begin{array}{l} u_{i}^{k}={\hat{u}}_{i}^{k}+n_{u}\\ v_{i}^{k}={\hat{v}}_{i}^{k}+n_{v} \end{array}$$

where $n_{u}$ and $n_{v}$ denote the measurement noises. They are assumed to follow zero-mean Gaussian distributions, $n_{u}\sim N(0,\sigma_{n_{u}}^{2})$, $n_{v}\sim N(0,\sigma_{n_{v}}^{2})$.

The center of a two-dimensional bounding box is only an approximate projection of the three-dimensional target center on the camera image plane. When the target is closer to the camera, its bounding box width $W_{i}$ and height $H_{i}$ in the image become larger. In this case, the target boundary is usually more blurred and is more easily affected by changes in viewing angle and partial occlusion. Therefore, a geometric uncertainty term related to the bounding-box size is introduced as

(35) $$\sigma_{u,scale}^{2}=\alpha W_{i}^{2},\;\sigma_{v,scale}^{2}=\beta H_{i}^{2},$$

where α and β are proportional coefficients of the scale-related uncertainty. By combining the detection noise of the target detection network with the physical scale-related noise, and assuming that the two terms are independent, the covariance matrix of the target bounding-box center pixel observation $\Sigma_{uv}$ can be modeled as

(36) $$\Sigma_{uv}=\left[\begin{array}{cc} \sigma_{n_{u}}^{2}+\sigma_{u,scale}^{2} & 0\\ 0 & \sigma_{n_{v}}^{2}+\sigma_{v,scale}^{2} \end{array}\right]=\left[\begin{array}{cc} \sigma_{n_{u}}^{2}+\alpha W_{i}^{2} & 0\\ 0 & \sigma_{n_{v}}^{2}+\beta H_{i}^{2} \end{array}\right],$$

Accordingly, the covariance component corresponding to the pixel perturbation terms $u_{i}^{k}$ and $v_{i}^{k}$ in Equation (27) is given by

(37) $$\Sigma_{pix}^{sem}=J_{u}\left(\sigma_{u}^{2}+\alpha W_{i}^{2}\right)J_{u}^{T}+J_{v}\left(\sigma_{v}^{2}+\beta H_{i}^{2}\right)J_{v}^{T},$$

(2) Depth error, camera pose error, and landmark prior error modeling:

Let the variance of the depth estimation error be $\sigma_{d}^{2}$. The covariance component corresponding to the depth perturbation term $d_{i}^{k}$ in Equation (33) is

(38) $$\Sigma_{dep}^{sem}=J_{d}\sigma_{d}^{2}J_{d}^{T},$$

where $\sigma_{d}^{2}$ denotes the variance of the depth estimation error. The covariance matrix of the camera pose error is denoted as

(39) $$\Sigma_{pose}=\left[\begin{array}{cc} \Sigma_{tt} & \Sigma_{t\theta }\\ \Sigma_{\theta t} & \Sigma_{\theta \theta } \end{array}\right],$$

where $\Sigma_{tt}$ denotes the translational error covariance, $\Sigma_{\theta \theta }$ denotes the rotational error covariance, and $\Sigma_{t\theta }$, $\Sigma_{\theta t}$ denote the cross-covariance between translation and rotation. The covariance component induced by the camera pose perturbation term $\xi_{c_{k}}^{w}$ in Equation (27) can be written as

(40) $$\begin{array}{ll} \Sigma_{pose}^{sem} & =J_{pose}\Sigma_{pose}J_{pose}^{T}\\ & =\Sigma_{tt}-\Sigma_{t\theta }{\left[d_{i}^{k}K^{-1}\left[\begin{array}{c} u_{i}^{k}\\ v_{i}^{k}\\ 1 \end{array}\right]\right]}_{\times }^{T}R_{c_{k}}^{wT}-R_{c_{k}}^{w}{\left[d_{i}^{k}K^{-1}\left[\begin{array}{c} u_{i}^{k}\\ v_{i}^{k}\\ 1 \end{array}\right]\right]}_{\times }\Sigma_{\theta t},\\ & +R_{c_{k}}^{w}{\left[d_{i}^{k}K^{-1}\left[\begin{array}{c} u_{i}^{k}\\ v_{i}^{k}\\ 1 \end{array}\right]\right]}_{\times }\Sigma_{\theta \theta }{\left[d_{i}^{k}K^{-1}\left[\begin{array}{c} u_{i}^{k}\\ v_{i}^{k}\\ 1 \end{array}\right]\right]}_{\times }^{T}R_{c_{k}}^{wT,} \end{array}$$

The prior coordinate error covariance of the fixed semantic landmark is denoted as

(41) $$\Sigma_{lm}=\left[\begin{array}{ccc} \sigma_{x}^{2} & 0 & 0\\ 0 & \sigma_{y}^{2} & 0\\ 0 & 0 & \sigma_{z}^{2} \end{array}\right],$$

where $\sigma_{x}^{2}$, $\sigma_{y}^{2}$, and $\sigma_{z}^{2}$ denote the variances of the landmark prior coordinate errors along the X-, Y-, and Z-axes, respectively. Since $J_{lm}=-I$, the covariance component corresponding to the landmark prior error is

(42) $$\Sigma_{lm}^{sem}=J_{lm}\Sigma_{lm}J_{lm}^{T}=\Sigma_{lm},$$

(3) Noise covariance derivation of the semantic factor:

According to Equations (32), (33), (35) and (37), the covariance matrix of the semantic factor can be expressed as:

(43) $$\begin{array}{l} \Sigma_{k}^{sem}=J_{u}\left(\sigma_{u}^{2}+\alpha W_{i}^{2}\right)J_{u}^{T}+J_{v}\left(\sigma_{v}^{2}+\beta H_{i}^{2}\right)J_{v}^{T}\\ +J_{d}\sigma_{d}^{2}J_{d}^{T}+J_{pose}\Sigma_{pose}J_{pose}^{T}+\Sigma_{lm,} \end{array}$$

To write different error sources in a unified block form, the joint perturbation vector is defined as

(44) $$\delta z_{sem}={\left[\begin{array}{cccccc} \delta u_{i}^{k} & \delta v_{i}^{k} & \delta d_{i}^{k} & \delta t_{c_{k}}^{wT} & \delta \theta_{c_{k}}^{wT} & \delta P_{i}^{wT} \end{array}\right]}^{T},$$

The corresponding joint covariance matrix is defined as

(45) $$\Sigma_{z}^{sem}=\mathrm{diag}\left(\Sigma_{uv},\;\sigma_{d}^{2},\;\Sigma_{pose},\;\Sigma_{lm}\right),$$

The Jacobian matrix of the semantic factor is defined as

(46) $$J_{sem}=\left[\begin{array}{cccccc} J_{u} & J_{v} & J_{d} & I_{3\times 3} & -R_{c_{k}}^{w}{\left[d_{i}^{k}K^{-1}\left[\begin{array}{c} u_{i}^{k}\\ v_{i}^{k}\\ 1 \end{array}\right]\right]}_{\times } & -I_{3\times 3} \end{array}\right],$$

Thus, the covariance of the semantic factor can also be written in the compact form as

(47) $$\Sigma_{k}^{sem}=J_{sem}\Sigma_{z}^{sem}J_{sem}^{T},$$

The parameter settings used for the semantic factor covariance model are listed in Table 1. Considering the detection confidence, the confidence-weighted semantic covariance is obtained according to Equation (30), and the semantic factor cost term is finally constructed as Equation (31). By substituting Equation (31) into the global objective function, the semantic factor is incorporated into the optimization problem shown in Equation (32).

## 4. Vehicle Experiment Validation and Analysis

**Figure 10:**
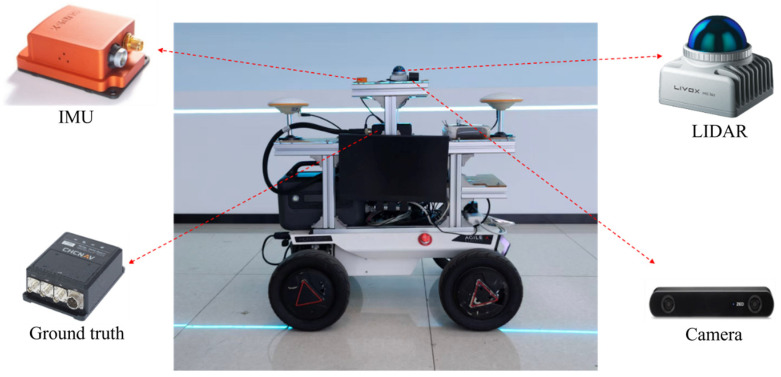
Vehicle experimental platform.

The unmanned ground vehicle experimental platform built in this study is shown in Figure 10. The platform mainly consists of a HUNTER unmanned vehicle chassis, an NVIDIA Jetson Orin Nano onboard computer, a ZED 2i stereo camera, a RoboSense Helios 32-line LiDAR, and an XSENS MTI-G-710 Inertial Measurement Unit (IMU). A CGI-430 high-precision tightly coupled navigation system is used to provide the reference pose for performance evaluation. The main parameters of the sensors are listed in Table 2.

### 4.1. KITTI Dataset Experiment Analysis

**Figure 11:**
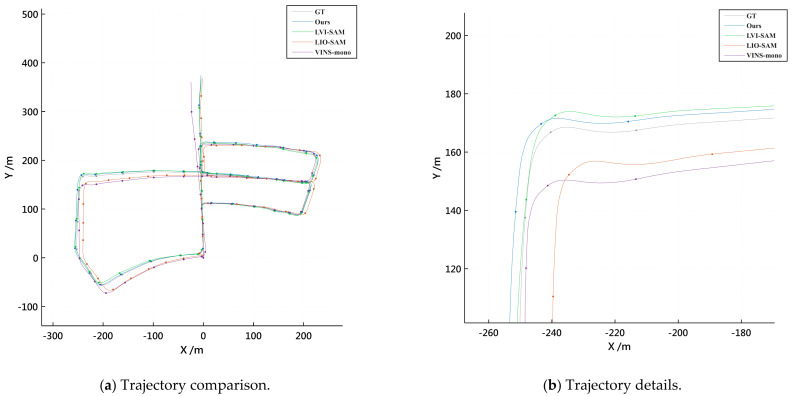
Comparison of trajectories estimated by different algorithms with the ground truth on KITTI 05.

To evaluate the performance of the proposed multi-source fusion framework and to provide a comparison with existing SLAM and multi-source fusion methods, an additional experiment was conducted on the KITTI 05 sequence. In this experiment, the proposed method was compared with several representative open-source localization and SLAM frameworks, including LVI-SAM, LIO-SAM, and VINS-Mono. The comparison mainly focuses on the trajectory consistency with the ground truth and the positioning error statistics. Figure 11 shows the trajectory comparison between different algorithms and the ground truth on KITTI 05. The black curve represents the ground truth trajectory, while the blue, green, red, and purple curves represent the trajectories estimated by the proposed method, LVI-SAM, LIO-SAM, and VINS-Mono, respectively. It can be observed from the global trajectory comparison in Figure 11a that the proposed method maintains good consistency with the ground truth throughout most of the route. LIO-SAM and VINS-Mono show more obvious deviations in several road sections, especially near turning and curved segments. This is mainly because VINS-Mono relies only on visual-inertial information and is more susceptible to accumulated visual drift, while LIO-SAM depends mainly on LiDAR-inertial constraints and may still be affected by local geometric degeneration. The enlarged trajectory details are shown in Figure 11b. In this local region, the proposed method follows the ground truth more closely than LIO-SAM and VINS-Mono. Compared with LVI-SAM, the proposed method also shows a similar trajectory trend and maintains competitive local consistency. This indicates that the proposed factor graph framework can effectively integrate multi-source constraints and provide stable trajectory estimation.

The quantitative positioning error statistics are listed in Table 3. The proposed method achieves a maximum error of 3.920568 m, which is lower than that of LVI-SAM, LIO-SAM, and VINS-Mono. The RMSE of the proposed method is 1.943426 m, which is close to that of LVI-SAM and significantly lower than those of LIO-SAM and VINS-Mono. Although LVI-SAM obtains slightly lower mean and median errors in this experiment, the proposed method shows a smaller maximum error, indicating better suppression of large local deviations. In contrast, LIO-SAM and VINS-Mono have larger RMSE, mean, and median errors, showing that their trajectories deviate more significantly from the ground truth in this sequence.

It should be noted that this KITTI experiment was conducted without introducing semantic factors. Therefore, the comparison mainly evaluates the basic multi-source fusion capability of the proposed framework. The results show that, even without semantic constraints, the proposed framework can achieve trajectory accuracy comparable to LVI-SAM and clearly better than LIO-SAM and VINS-Mono in this experiment. This demonstrates that the proposed backend factor graph optimization and factor quality control strategy can provide a reliable localization foundation. On this basis, the semantic factors introduced in the vehicle-mounted experiments further provide supplementary global constraints under GNSS-degraded conditions, thereby improving the long-term positioning robustness of the system.

### 4.2. Vehicle-Based Test Scenario

The experimental validation consists of two vehicle-mounted experiments. Experiment 1 has a total driving time of approximately 360 s, and two fixed semantic landmarks are placed along the route. This test scenario can simulate the operating conditions of a multi-source integrated navigation system in a vehicle-mounted environment. The experiment is conducted under degraded GNSS positioning conditions to verify the proposed factor quality control method and semantic factor.

To further evaluate the applicability of the proposed method in more complex scenarios, Experiment 2 was conducted on a longer route with more complicated environmental conditions. The route includes ordinary road sections, tree-covered areas, building-occluded areas, and an industrial park environment, covering several typical degradation scenarios encountered in practical applications. The data were collected under strong midday illumination, where image observations are more likely to be affected by illumination variations and local overexposure. In addition, a longer GNSS-degraded interval was designed in Experiment 2, and the route also includes several sections that are unfavorable for LiDAR to stably extract geometric features. These factors further increase the difficulty of multi-source fusion localization in complex environments.

**Figure 12:**
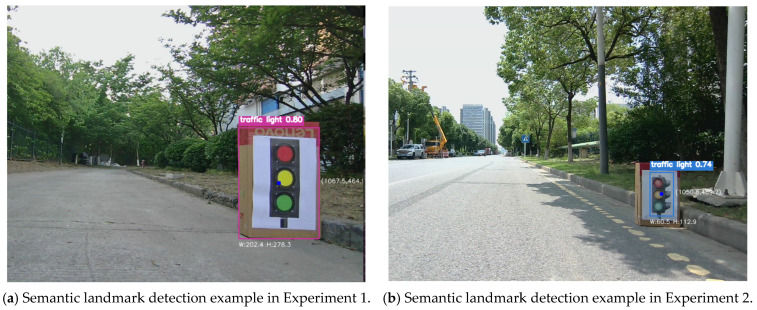
Semantic landmark detection results in the experimental scene.

In both experiments, traffic signal lights are arranged as semantic landmarks at specific positions along the experimental route. Figure 12 shows representative semantic landmark detection results in the two experiments. Specifically, Figure 12a shows the semantic landmark detection example in Experiment 1, and Figure 12b shows the semantic landmark detection example in Experiment 2. The information output by the semantic detection module mainly includes the target category, the center pixel coordinate $p_{i}^{k}$ of the detection box, and the detection confidence $s_{i}^{k}$. The target category is used to determine whether the detection result belongs to the pre-arranged fixed semantic landmark. The center pixel coordinate of the detection box and the depth information are used to recover the three-dimensional position of the semantic landmark in the camera frame. The detection confidence reflects the reliability of the current recognition result and is used in the subsequent weighting of the semantic factor. In addition, the known position $P_{i}^{w}$ of each semantic landmark in the world frame is stored in advance by the system.

### 4.3. Vehicle-Based Experimental Evaluation of Semantic Factors

#### 4.3.1. Experimental Results and Analysis of Experiment 1

The prior positions of the fixed semantic landmarks were determined before the experiment. Specifically, two fixed traffic lights near the intersection on the lower-left side of the experimental trajectory were selected as semantic landmarks. Their latitude, longitude, and altitude were measured by RTK and then transformed into the local world frame used in the experiment. The approximate coordinates of the two semantic landmarks in the local world frame were (−40.479, −83.314, −0.192) m and (−43.066, −80.127, −0.223) m, respectively. During the experiment, the two landmarks could be observed by the camera during 249–265 s and 255–270 s, respectively. Semantic factors were constructed only when the corresponding landmark was successfully detected and passed the confidence, depth validity, and landmark association checks.

The middle stage of the experiment, from 200 s to 300 s, was designed to simulate GNSS degradation in practical applications. To simulate GNSS degradation, the covariance of the GNSS factor was enlarged to reduce its weight during this period. Under normal conditions, the standard deviation of the GNSS position factor was set to 0.1 m. During the GNSS-degraded interval, the standard deviation of the GNSS position factor was increased to 10 m. Therefore, the GNSS factor was not completely removed during this interval, but participated in the backend optimization with a significantly reduced weight. This setting was used to simulate the condition in which GNSS measurements are still available but their reliability is significantly degraded due to building blockage and multipath effects.

This strategy provides a controllable and repeatable way to evaluate the influence of degraded GNSS constraints on the proposed factor graph optimization framework. However, it does not fully represent real GNSS outages, severe multipath distortion, or measurements collected in urban canyon environments. In real urban scenarios, GNSS degradation may involve complete signal interruption, intermittent availability, non-Gaussian errors, and complex multipath effects. Therefore, experiments using real GNSS outages and urban canyon datasets will be further investigated in future work.

**Figure 13:**
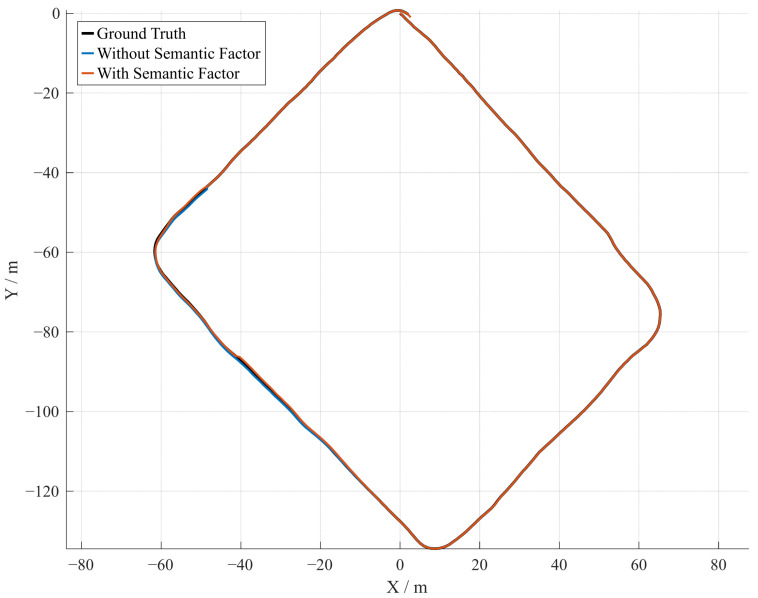
Comparison of the estimated trajectory and ground truth in Experiment 1.

In Figure 13, the black curve represents the reference ground truth, the blue curve represents the trajectory without semantic factors, and the orange curve represents the trajectory estimated by the algorithm with semantic factors. It can be observed that, in the region where GNSS signals are disturbed, the trajectory with semantic factors achieves higher positioning accuracy than the trajectory without semantic factors. This indicates that the absolute reference information provided by fixed semantic landmarks can effectively impose global constraints on the system trajectory under GNSS-degraded conditions, thereby reducing the influence of accumulated errors from other sensors on the overall positioning result.

**Figure 14:**
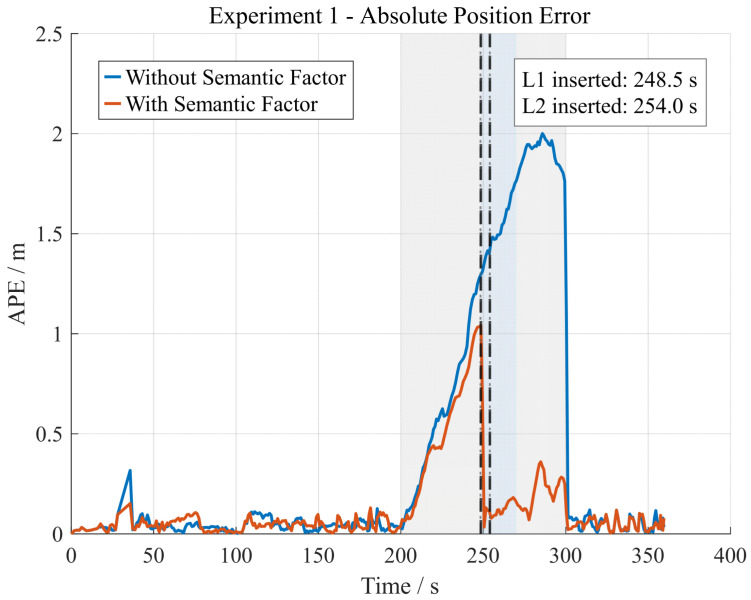
Comparison of absolute trajectory error with and without semantic factors in Experiment 1.

From the absolute pose error (APE) curves shown in Figure 14, it can be seen that the APE values of both methods remain at a low level in the first half of the experiment, indicating that the system can maintain stable positioning performance when GNSS constraints are effective and multi-source observations are reliable. As marked by the shaded region in Figure 14, the interval from 200 s to 300 s corresponds to the GNSS-degraded period. After entering this interval, the error of the algorithm without semantic factors increases significantly. During the period from 200 s to 300 s, the APE curve shows a continuous upward trend and reaches a peak of about 2 m in the later part of this interval. This suggests that, in the absence of sufficiently reliable global absolute constraints, the integrated navigation system based on IMU, LiDAR, and visual information is prone to accumulated drift.

In contrast, for the proposed method, the two vertical dashed lines in Figure 14 indicate the first insertion times of the semantic factors constructed from the two semantic landmarks into the backend factor graph optimization, which are approximately 248.5 s and 254.0 s, respectively. After these semantic factors are introduced, the error growth is effectively suppressed, and the APE remains consistently lower than that of the method without semantic factors. This demonstrates that fixed semantic landmarks can provide additional global position constraints when GNSS constraints are insufficient, thereby suppressing drift and improving the stability of the positioning results.

**Figure 15:**
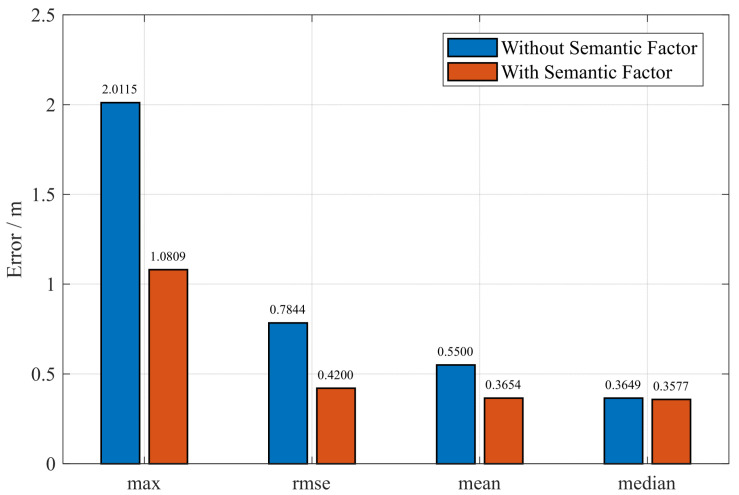
Bar chart of absolute trajectory errors in Experiment 1.

Figure 15 shows the statistical bar chart of the absolute trajectory error. After semantic factors are introduced, the maximum error decreases from 2.0115 m to 1.0809 m, corresponding to a reduction of approximately 46.26%. The root mean square error decreases from 0.7544 m to 0.4200 m, with a reduction of approximately 44.32%. The mean error decreases from 0.5500 m to 0.3654 m, corresponding to a reduction of approximately 33.56%. These results indicate that, in the absence of GNSS absolute constraints, semantic factors can effectively suppress overall trajectory drift and significantly improve the error distribution of the system.

Table 4 provides a quantitative analysis of the three-axis position error statistics. It can be seen that, after semantic factors are introduced, the position errors along the X, Y, and Z-axes are significantly improved. For the *X*-axis, the RMSE decreases from 0.1720 m to 0.0952 m, corresponding to a reduction of approximately 44.65%, and the maximum error decreases from 0.6174 m to 0.3487 m, corresponding to a reduction of approximately 43.52%. For the *Y*-axis, after introducing semantic factors, the RMSE decreases from 0.1201 m to 0.0749 m, with a reduction of approximately 37.64%, and the maximum error decreases from 0.4405 m to 0.3001 m, with a reduction of approximately 31.87%. For the *Z*-axis, the RMSE decreases from 0.6995 m to 0.1730 m, corresponding to a reduction of approximately 75.27%; the maximum error decreases from 1.9353 m to 0.9035 m, corresponding to a reduction of approximately 53.32%; and the mean error decreases from 0.3290 m to 0.0619 m.

Overall, under GNSS-degraded conditions, the introduction of semantic factors significantly improves the trajectory error and the three-axis position errors of the system. The maximum absolute trajectory error is reduced by approximately 46.26%. These results indicate that fixed semantic landmarks can provide effective global constraints for the system, and that using fixed prior semantic landmarks as global constraints in multi-source integrated navigation has practical value.

#### 4.3.2. Experimental Results and Analysis of Experiment 2

To further verify the applicability of the proposed method in more complex scenarios, Experiment 2 was conducted on a longer route with more complicated environmental conditions. The route includes ordinary road sections, tree-lined areas, building-occluded areas, and an industrial park environment, covering several typical degradation scenarios encountered in practical applications. The data were collected under strong midday illumination, where image observations are more likely to be affected by illumination variations and local overexposure.

In addition, a longer and more severe GNSS-degraded interval was designed in this experiment, and the route also includes several sections that are unfavorable for LiDAR to stably extract geometric features. Under normal conditions, the standard deviation of the GNSS position factor was set to 0.1 m. In Experiment 1, it was increased to 10 m during the GNSS-degraded interval. In Experiment 2, the standard deviation of the GNSS position factor was further increased to 30 m during the degraded interval, so that the GNSS factor was assigned a lower weight than that in Experiment 1. Therefore, Experiment 2 was used to evaluate the proposed method under a longer-duration and more severe GNSS-degraded condition. These factors further increase the difficulty of multi-source fusion localization in complex environments.

In this experiment, the GNSS-degraded interval was set from 100 s to 430 s. Meanwhile, 6 prior semantic landmarks were added along the experimental route, with an average lateral distance of approximately 2.5–4 m from the vehicle trajectory. Landmark association was performed based on depth consistency. Specifically, each prior semantic landmark was first transformed into the current camera frame using the predicted vehicle pose, and its predicted depth was then calculated. The measured depth was extracted from the local depth window around the detected bounding-box center. The detected traffic light was associated with the candidate landmark whose predicted depth was closest to the measured depth. If the depth difference exceeded the predefined threshold of 0.5 m, the semantic observation was rejected. The corresponding semantic factors were inserted into the backend factor graph at approximately 148 s, 180 s, 249 s, 294 s, 370 s, and 389 s, respectively.

**Figure 16:**
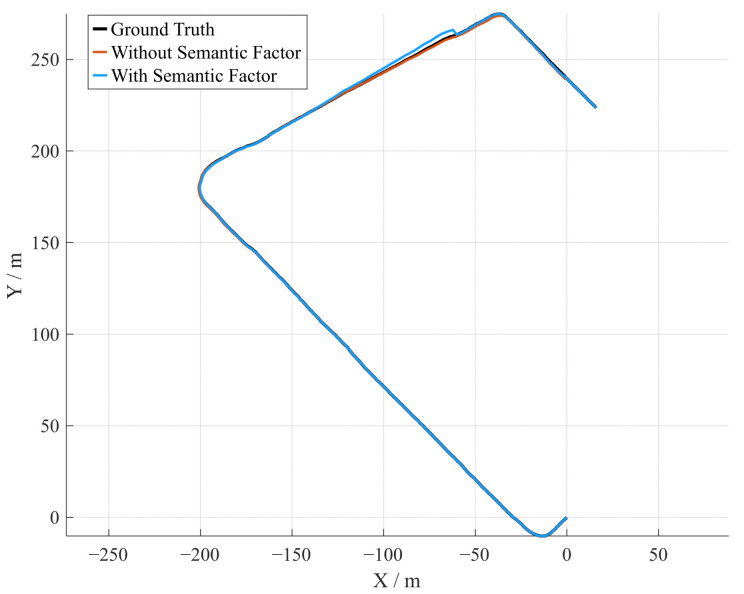
Comparison of the estimated trajectory and ground truth in Experiment 2.

Figure 16 shows the trajectory comparison of the reference trajectory, the method without semantic factors, and the proposed method with semantic factors. It can be observed that the route in this experiment is more complicated than that in the previous experiment, involving a longer travel distance and more complex motion changes. During the GNSS-degraded interval, the trajectory estimated without semantic factors gradually deviates from the reference trajectory, especially in the middle and later parts of the route. In contrast, the trajectory estimated by the proposed method remains closer to the reference trajectory. This indicates that when multiple semantic landmarks are available, the proposed semantic factors can provide repeated global constraints along the route and help correct the accumulated drift of the multi-source integrated navigation system. 

**Figure 17:**
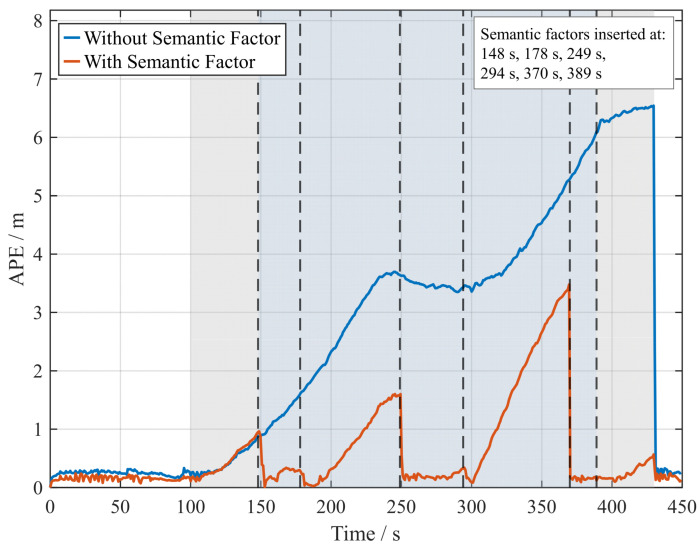
APE comparison with and without semantic factors in Experiment 2.

The APE comparison is shown in Figure 17. The shaded region represents the GNSS-degraded interval from 100 s to 430 s, and the vertical dashed lines indicate the first insertion times of semantic factors. As shown in the figure, after entering the GNSS-degraded interval, the APE of the method without semantic factors gradually increases and finally reaches a peak of more than 6 m. This shows that, under long-term GNSS degradation, the accumulated error of the system becomes significant when only IMU, LiDAR, and visual constraints are used. In contrast, the proposed method effectively suppresses the error growth after semantic factors are introduced. Although the error may still increase between two adjacent semantic observations, it is significantly corrected after new semantic factors are inserted into the factor graph. This phenomenon is particularly evident near the semantic factor insertion times of 148 s, 249 s, 294 s, and 370 s, where the APE of the proposed method is reduced or maintained at a lower level compared with the method without semantic factors.

**Figure 18:**
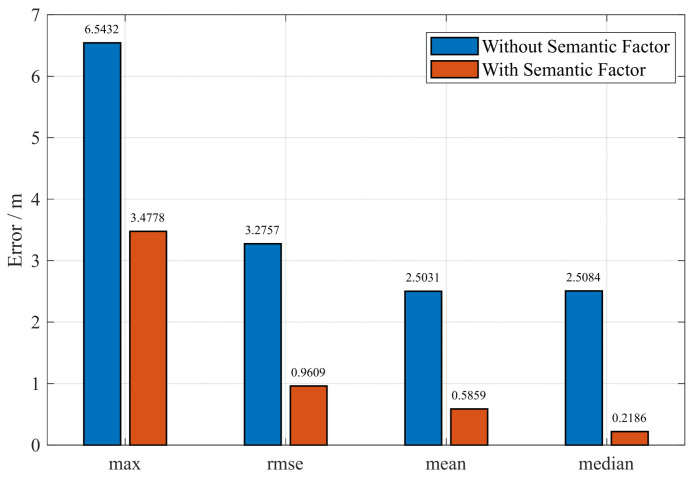
Bar chart of APE statistics in Experiment 2.

To further quantitatively evaluate the positioning accuracy in Experiment 2, the APE statistical results are shown in Figure 18. Compared with the method without semantic factors, the proposed method reduces the maximum APE from 6.5432 m to 3.4778 m, the RMSE from 3.2757 m to 0.9609 m, the mean error from 2.5031 m to 0.5859 m, and the median error from 2.5084 m to 0.2136 m. The RMSE, mean, and median errors are significantly reduced after introducing semantic factors, indicating that the proposed method can effectively suppress accumulated drift during long-term GNSS degradation. Although the maximum error is still affected by local complex environments and intermittent semantic landmark visibility, the overall error distribution is greatly improved. These results further demonstrate that multiple fixed semantic landmarks can provide effective global constraints for the backend factor graph and improve the robustness of multi-source fusion localization on more complicated routes.

This experiment demonstrates that the proposed method is not limited to a single route or two semantic landmarks. Therefore, multiple semantic landmarks can provide intermittent but effective global position constraints under GNSS-degraded conditions. The results further verify that the proposed method can improve localization robustness on more complicated routes with multiple landmarks and long-duration GNSS degradation.

**Figure 19:**
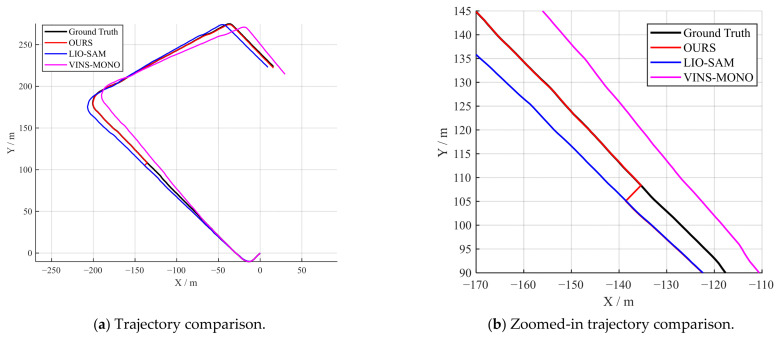
Trajectory comparison among the proposed methods LIO-SAM and VINS-MONO.

In Figure 19a, the proposed method is compared with two representative baseline methods, LIO-SAM and VINS-MONO, using the same ground-truth trajectory as the reference. Figure 19b shows the zoomed-in trajectory details. VINS-MONO exhibits larger trajectory drift, while LIO-SAM achieves better trajectory continuity than VINS-MONO. However, when relying only on geometric constraints, LIO-SAM still shows local deviations. In contrast, the proposed method introduces semantic factors as supplementary global constraints under GNSS-degraded conditions, making the estimated trajectory closer to the ground truth.

**Figure 20:**
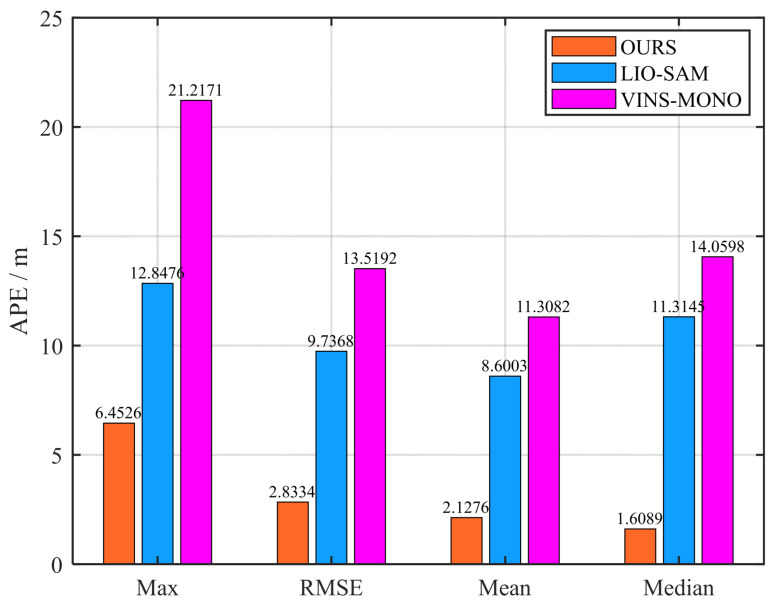
APE statistics of the proposed method, LIO-SAM, and VINS-MONO.

The APE statistical results are shown in Figure 20. The proposed method achieves the lowest errors among all four statistical metrics. Specifically, the maximum APE of the proposed method is 6.4526 m, whereas those of LIO-SAM and VINS-MONO are 12.8476 m and 21.2171 m, respectively. The RMSE of the proposed method is 2.8334 m, which is significantly lower than 9.7368 m for LIO-SAM and 13.5192 m for VINS-MONO. Compared with LIO-SAM, the proposed method reduces the maximum error and RMSE by 49.78% and 70.90%, respectively. Compared with VINS-MONO, the corresponding reductions are 69.59% and 79.04%, respectively. In addition, the mean and median errors are also significantly reduced, indicating that the proposed method not only reduces the peak error but also improves the overall trajectory consistency. These results further demonstrate the effectiveness of incorporating semantic factors into the multi-source factor graph optimization framework.

### 4.4. Scalability Verification Using Natural Urban Semantic Landmarks

**Figure 21:**
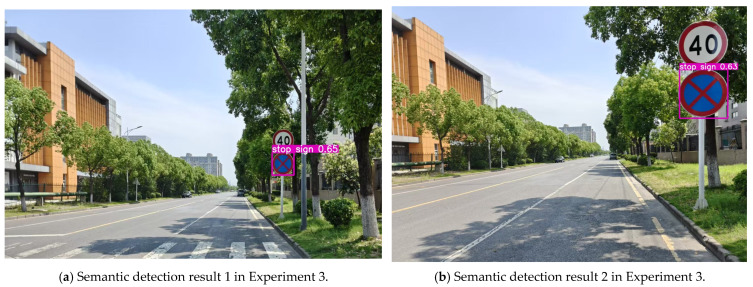
Detection results of stop-sign semantic landmarks in the experimental scene.

To further evaluate the scalability of the proposed semantic-enhanced localization method beyond pre-prepared traffic-light landmarks, an additional Experiment 3 using naturally existing urban semantic features was conducted on the second experimental trajectory. As shown in Figure 21, traffic signs that already existed in the road environment were selected as semantic landmarks. These traffic signs were not manually arranged for the experiment, but were natural roadside semantic objects observed by the vehicle-mounted camera during driving. The YOLOv8n-based detector successfully identified these traffic-sign landmarks in real road-scene images, and their global coordinates were measured in advance to provide prior landmark information for semantic factor construction.

**Figure 22:**
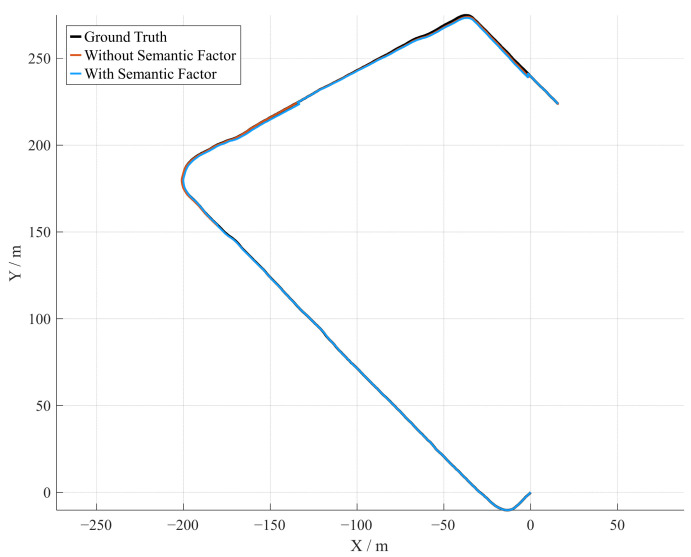
Comparison of the estimated trajectory and ground truth in Experiment 3.

**Figure 23:**
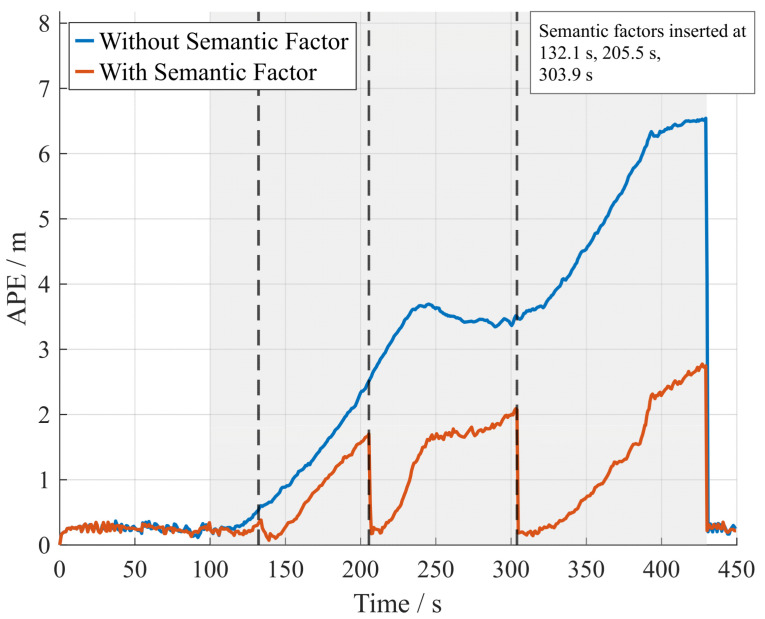
APE comparison with and without semantic factors in Experiment 3.

Based on the detected traffic-sign landmarks, semantic factors were inserted into the factor graph at 132.1 s, 205.5 s, and 303.9 s. Figure 22 shows the trajectory comparison with and without semantic factors, and Figure 23 presents the corresponding absolute position error (APE). It can be observed that, without semantic factors, the estimated trajectory gradually deviates from the ground truth during the GNSS-degraded interval, and the APE increases continuously. After introducing semantic factors constructed from the naturally existing traffic signs, the accumulated trajectory error is effectively suppressed.

Quantitatively, the maximum trajectory error is reduced from 6.5407 m to 2.7737 m, corresponding to a reduction of 57.59%. The RMSE is reduced from 3.2912 m to 1.1607 m, corresponding to a reduction of 64.73%. These results indicate that semantic factors constructed from natural urban semantic landmarks can provide effective supplementary global constraints under GNSS-degraded conditions.

This experiment further demonstrates that the proposed method is not limited to pre-prepared traffic-light landmarks. When stable semantic objects in urban environments can be detected, and their prior global coordinates are available, they can also be incorporated into the factor graph as semantic constraints. Therefore, the proposed method can be extended to naturally existing urban landmark features.

### 4.5. Real-Time Performance Analysis

The computational efficiency of the proposed framework was evaluated on the onboard embedded computer, i.e., NVIDIA Jetson Orin Nano. Since the Jetson Orin Nano is a resource-constrained embedded platform, the real-time performance of the complete system was analyzed according to the runtime of the main modules.

In the implemented system, the IMU, camera, and LiDAR operated at 100 Hz, 10 Hz, and 10 Hz, respectively. For semantic detection, the lightweight YOLOv8n model was adopted, and only one object category, i.e., traffic light, was detected. This single-class detection setting reduced the computational burden of network inference and post-processing. The YOLOv8n detection module was triggered by camera frames, and its effective detection frequency was approximately 10 Hz. The average inference time was approximately 25 ms per frame on the Jetson Orin Nano, which was lower than the 100 ms frame interval of the 10 Hz camera stream. Therefore, the semantic detection module could satisfy the real-time requirement of the image stream.

The semantic factor construction was event-triggered and was executed only when valid traffic-light observations were available. The 2D-to-3D coordinate inversion and covariance propagation involved only coordinate transformation and basic Jacobian matrix operations, and the additional computation time was less than 1 ms per valid semantic observation. The backend factor graph optimization took approximately 15 ms per update, which was also lower than the 100 ms update period corresponding to the 10 Hz camera/LiDAR input.

During the experiment, 3678 backend-optimized pose records were generated over approximately 367 s, corresponding to an average localization output rate of about 10.02 Hz. This output rate is consistent with the 10 Hz camera/LiDAR input frequency. Therefore, the complete system can process semantic detection, semantic factor construction, and backend optimization within the main sensor update interval, indicating that the proposed method can run in real time on the onboard Jetson Orin Nano platform.

### 4.6. Discussion on Failure Cases and Limitations

Although the proposed semantic factor can provide supplementary global constraints under GNSS-degraded conditions, its performance still depends on the reliability of semantic detection, depth estimation, landmark association, and prior landmark coordinates. Therefore, several potential failure cases and limitations should be considered in practical applications.

First, false detections may introduce incorrect semantic observations. Since the semantic factor is used as an additional absolute position constraint in the backend factor graph, a false detection may introduce a biased global constraint and directly affect the state estimation. Therefore, the proposed method is more sensitive to false positives than to false negatives. In contrast, a missed detection only means that no semantic factor is constructed at the current time. In this case, the system falls back to the original multi-source integrated navigation framework and continues to rely on IMU, GNSS, LIO, and VIO factors. For this reason, a relatively conservative confidence threshold is adopted in this paper. Only detections with confidence scores higher than 0.5 are accepted for semantic factor construction, while detections below this threshold are rejected before being inserted into the factor graph. For accepted detections, the confidence score is further used to adjust the semantic factor covariance, so that observations with lower confidence are assigned larger covariance and smaller weight in the backend optimization.

Second, in the current implementation, landmark association is performed based on depth consistency. Specifically, each prior semantic landmark is first transformed into the current camera frame using the predicted vehicle pose, and its predicted depth is then calculated. The measured depth is extracted from the local depth window around the detected bounding-box center. The detected traffic light is associated with the candidate landmark whose predicted depth is closest to the measured depth. If the depth difference exceeds the predefined threshold of 0.5 m, the semantic observation is rejected. This strategy can reduce the risk of incorrect association when candidate landmarks have distinguishable depths. In future work, image-plane projection gating and temporal semantic tracking will be introduced to further improve the robustness of landmark association.

Third, depth errors may occur because traffic lights are relatively small objects, and stereo depth estimation can be noisy or invalid around object boundaries. To improve robustness, the depth value is extracted from a local window centered at the bounding-box center rather than from a single pixel. Invalid depth values are removed, and the median value of valid depth pixels is used for 3D back-projection.

Fourth, partial occlusion, strong illumination changes, and long observation distances may reduce detection confidence and degrade the accuracy of the bounding-box center. In these cases, the semantic factor covariance is enlarged through the confidence-weighted covariance model, thereby reducing the influence of uncertain semantic observations on the final state estimation.

Fifth, when no semantic landmark is visible, no semantic factor is constructed. In this case, the system degenerates to the original multi-source integrated navigation framework composed of IMU, LiDAR, visual odometry, and GNSS factors. Therefore, the proposed semantic factor acts as a supplementary global constraint rather than a mandatory observation source.

Finally, inaccurate prior coordinates of semantic landmarks may introduce biased absolute constraints. To account for this uncertainty, the landmark prior coordinate error is explicitly included in the semantic factor covariance model. In real applications, the prior coordinates should be obtained using reliable surveying equipment, such as RTK static measurement.

## 5. Conclusions

To address the loss of global constraints caused by GNSS signals being affected by building blockage, multipath effects, and other factors in complex urban environments, a multi-sensor fusion method incorporating semantic information within a factor graph optimization framework is proposed.

(1) Measurement models for multi-source sensors, including GNSS, IMU, LiDAR, and cameras, are established. The sensor data are preprocessed, and the visual SLAM and LiDAR SLAM subsystems are introduced. Then, IMU preintegration factors, GNSS factors, LiDAR odometry factors, and visual odometry factors are constructed, enabling observations from different sources to be fused within a unified backend optimization framework.

(2) Different from conventional methods that mainly rely on geometric observations, fixed traffic lights are selected as semantic landmarks in this paper. An object detection network is used to extract the center pixel coordinates and confidence scores of semantic landmarks from images. By combining depth estimation, camera pose, and the prior global coordinates of fixed semantic landmarks, a semantic target three-dimensional inversion model is established. In this way, local semantic observations in images are transformed into absolute constraint information in the world coordinate system, providing supplementary global constraints for the system.

(3) Experiments are conducted to validate the proposed framework. The KITTI 05 experiment compares the proposed method with representative SLAM and multi-source fusion methods, including LVI-SAM, LIO-SAM, and VINS-Mono. The results show that, even without introducing semantic factors, the proposed framework achieves trajectory accuracy comparable to LVI-SAM and clearly better than LIO-SAM and VINS-Mono in this experiment, demonstrating the effectiveness of the proposed multi-source fusion backend and factor quality control strategy.

Vehicle-mounted experiments are carried out to evaluate the effect of semantic factors under GNSS-degraded conditions. In Experiment 1, compared with the method without semantic factors, the maximum positioning errors along the three axes are reduced by 43.52%, 31.87%, and 53.32%, respectively, and the maximum absolute trajectory error is reduced by 46.26%. To further evaluate the applicability of the proposed method, Experiment 2 is conducted on a longer and more complicated route with six prior semantic landmarks and a longer GNSS-degraded interval. The results show that the proposed method reduces the maximum APE from 6.5432 m to 3.4778 m, the RMSE from 3.2757 m to 0.9609 m, the mean error from 2.5031 m to 0.5859 m, and the median error from 2.5084 m to 0.2136 m. The maximum absolute trajectory error is reduced by approximately 46.85%. These results demonstrate that the proposed semantic factor can effectively provide supplementary global constraints and suppress accumulated drift when GNSS constraints are degraded.

This paper selects fixed traffic lights as semantic landmarks and uses their prior global coordinates to construct semantic constraints. This method verifies the role of semantic factors in suppressing accumulated system errors. However, the types of semantic targets used in the experiment are relatively limited, and the method relies on manually arranged landmarks with pre-measured coordinates. In addition, GNSS degradation in the current experiments is modeled by increasing the covariance of the GNSS factor, which does not fully represent real GNSS outages, severe multipath distortion, or measurements in urban canyon environments. In future work, a wider range of semantic target types can be further explored, and semantic methods based on prior maps can be investigated. More realistic GNSS-degraded scenarios, including real GNSS outages, multipath effects, and urban canyon datasets, will also be tested to further evaluate the robustness of the proposed method. In addition, joint modeling of semantic confidence, semantic category consistency, and geometric consistency can be further studied to improve the reliability of semantic factors in complex environments.

## Figures and Tables

**Table 1:** Parameter settings of the semantic factor covariance model.

Parameter	Meaning	Value Used in the Experiment	Source
$\sigma_{n_{u}}^{2},\sigma_{n_{v}}^{2}$	Pixel localization noise variance	$1.0\;{\mathrm{pixel}}^{2}$	Empirical estimation based on detection results
α,β	Scale-related coefficient in horizontal/vertical pixel direction	$1.0\times 10^{-3}$	Empirical tuning
$\sigma_{d}^{2}$	Depth estimation variance	$0.09\;m^{2}$	Stereo camera calibration
$\sigma_{x}^{2},\sigma_{y}^{2},\sigma_{z}^{2}$	Landmark prior coordinate variance	$0.01\;m^{2}$	RTK static measurement accuracy
$\Sigma_{tt}$	Translational covariance of camera pose	$\mathrm{diag}(0.04,\;0.04,\;0.04)\;m^{2}$	Estimated from frontend odometry uncertainty
$\Sigma_{\theta \theta }$	Rotational covariance of camera pose	$\mathrm{diag}(0.04,\;0.04,\;0.04)\;m^{2}$	Estimated from frontend odometry uncertainty
$\Sigma_{t\theta },\Sigma_{\theta t}$	Translation-rotation cross covariance	$0_{3\times 3}$	Simplified setting

**Table 2:** Main parameters of the sensors.

Sensor	Specification	Parameter	Frequency
XSENS MTi-G-710	Gyroscope bias instability	10 deg/h	100 HZ
	Gyroscope noise density	0.01 º/s/√Hz	
	Accelerometer bias instability	15 µg	
	Accelerometer noise density	60 µg/√Hz	
Zed2i	Focal length	4 mm	10 HZ
	Maximum resolution	2208 × 1242	
	Depth range	1.5 m to 35 m	
Helios 32	Channels	32	10 HZ
	Measurement range	0.2 m to 150 m	

**Table 3:** Positioning error statistics of different algorithms.

Algorithm	Max (m)	RMSE (m)	Mean (m)	Median (m)
OURS	3.920568	1.943426	1.900634	1.750774
LVI-SAM	4.888965	1.918667	1.702914	1.467012
LIO-SAM	9.150342	3.562704	2.944053	2.328173
VINS-MONO	32.103099	13.465721	12.369641	11.245719

**Table 4:** Statistics of three-axis positioning errors.

Position Error/m	Method	RMSE	MAX	Median	Mean
*X*-axis	With Semantic Factor	0.0952	0.3487	0.0612	0.0339
	Without Semantic Factor	0.1720	0.6174	0.0976	0.0358
*Y*-axis	With Semantic Factor	0.0749	0.3001	0.0501	0.0294
	Without Semantic Factor	0.1201	0.4405	0.0701	0.0353
*Z*-axis	With Semantic Factor	0.1730	0.9035	0.0619	0.0035
	Without Semantic Factor	0.6995	1.9353	0.3290	0.0034

## Data Availability

Data are contained within the article. The data presented in this study can be requested from the authors.
